# Cortical thickness subtypes in cognitively unimpaired individuals: Differential network and transcriptomic vulnerability to cortical thinning

**DOI:** 10.1002/alz.70762

**Published:** 2025-10-14

**Authors:** Luigi Lorenzini, Mario Tranfa, Leonard Pieperhoff, Federico Masserini, Mara ten Kate, Lyduine E. Collij, Giuseppe Pontillo, Emma S. Luckett, Alle Meije Wink, Henk JMM Mutsaerts, Tiago Gil Oliveira, Daniele Altomare, Mercè Boada, Anouk den Braber, Cindy Birck, Christopher Buckley, Gill Farrar, Wiesje van der Flier, Giovanni B. Frisoni, Rossella Gismondi, Juan Domingo Gispert, Bernard J. Hanseeuw, Frank Jessen, Marta Marquié, Anja Mett, Craig Ritchie, Gemma Salvadó, Michael Schöll, Mahnaz Shekari, Andrew W. Stephens, Betty M. Tijms, David Vállez García, Rik Vandenberghe, Pieter Jelle Visser, Luca Roccatagliata, Neil P. Oxtoby, Matteo Pardini, Frederik Barkhof

**Affiliations:** ^1^ Department of Radiology and Nuclear Medicine Amsterdam University Medical Centre, Vrije Universiteit Amsterdam The Netherlands; ^2^ Department of Neuroscience, Rehabilitation, Ophthalmology, Genetics, Maternal and Child Health (DiNOGMI) University of Genoa Genoa Italy; ^3^ Department of Advanced Biomedical Sciences University “Federico II” Naples Italy; ^4^ Amsterdam Neuroscience Brain imaging Amsterdam the Netherlands; ^5^ Department of Biomedical and Clinical Sciences Neuroscience Research Center University of Milan Milan Italy; ^6^ Alzheimer Center Amsterdam Department of Neurology Amsterdam UMC Amsterdam The Netherlands; ^7^ Clinical Memory Research Unit Department of Clinical Sciences Malmö, Faculty of Medicine Lund University Lund Sweden; ^8^ Queen Square Multiple Sclerosis Centre, Department of Neuroinflammation UCL Queen Square Institute of Neurology University College London London UK; ^9^ Department of Anatomy and Neurosciences MS Center Amsterdam, Amsterdam Neuroscience, Amsterdam UMC, Vrije Universiteit Amsterdam Amsterdam the Netherlands; ^10^ Laboratory for Cognitive Neurology KU Leuven Leuven Belgium; ^11^ Laboratory for Complex Genetics KU Leuven Leuven Belgium; ^12^ Life and Health Sciences Research Institute (ICVS) School of Medicine University of Minho Braga Portugal; ^13^ ICVS/3B's–PT Government Associate Laboratory University of Minho Braga/Guimarães Portugal; ^14^ Competence Centre on Ageing (CCA); Department of Business Economics Health and Social Care (DEASS); University of Applied Sciences and Arts of Southern Switzerland (SUPSI) Manno Switzerland; ^15^ Ace Alzheimer Center Barcelona–Universitat Internacional de Catalunya Barcelona Spain; ^16^ Networking Research Center on Neurodegenerative Diseases (CIBERNED) Instituto de Salud Carlos III Madrid Spain; ^17^ Amsterdam Neuroscience Neurodegeneration Amsterdam the Netherlands; ^18^ Alzheimer Europe Alzheimer Europe Bertrange Luxembourg; ^19^ GE HealthCare GE HealthCare Amersham UK; ^20^ Department of Epidemiology & Biostatistics Amsterdam Neuroscience, Amsterdam Amsterdam The Netherlands; ^21^ Department of Rehabilitation and Geriatrics Geneva University Hospitals Geneva Switzerland; ^22^ Life Molecular Imaging Life Molecular Imaging Berlin Germania; ^23^ Barcelonaβeta Brain Research Center Pasqual Maragall Foundation Barcelona Spain; ^24^ Department of Neurology Cliniques Universitaires Saint‐Luc Bruxelles Belgio; ^25^ Institute of Neuroscience Université catholique de Louvain Brussels Belgium; ^26^ Department of Psychiatry and Psychotherapy University Hospital Cologne Cologne Germany; ^27^ Scottish Brain Sciences Scottish Brain Sciences Edinburgh Scotland; ^28^ Department of Psychiatry and Neurochemistry Institute of Neuroscience and Physiology The Sahlgrenska Academy, University of Gothenburg Gothenburg Sweden; ^29^ Wallenberg Centre for Molecular and Translational Medicine University of Gothenburg Gothenburg Sweden; ^30^ Dementia Research Centre, Institute of Neurology University College London London UK; ^31^ Hospital del Mar Medical Research Institute (IMIM) PRBB Parc de Recerca Biomèdica de Barcelona Barcelona Spain; ^32^ Leuven Brain Institute KU Leuven Leuven Belgium; ^33^ Department of Psychiatry Maastricht University Maastricht the Netherlands; ^34^ Department of Health Sciences (DISSAL) University of Genoa Genoa Italy; ^35^ Department of Neuroradiology IRCCS Ospedale Policlinico San Martino Genoa Italy; ^36^ UCL Hawkes Institute and Department of Computer Science University College London London UK; ^37^ Clinica Neurologica IRCCS Ospedale Policlinico San Martino Genoa Italy; ^38^ Institutes of Neurology and Healthcare Engineering University College London London UK

**Keywords:** biological pathways, magnetic resonance imaging, polygenic risk, preclinical Alzheimer's

## Abstract

**INTRODUCTION:**

The emergence, stability, and contributing factors of Alzheimer's disease (AD) gray matter subtypes remain unclear.

**METHODS:**

We analyzed data from 1323 individuals without a diagnosis of dementia (CDR < 1) with T1w‐MRI and amyloid‐PET, including 622 with longitudinal data (3.66 ± 1.78 years). Cortical thickness subtypes were identified using a non‐negative matrix factorization (NMF) clustering algorithm. We examined clinical and demographic differences, subtype stability, and longitudinal thinning patterns using brain network models and imaging‐transcriptomic analysis. Replication was performed with an alternative clustering approach and a validation cohort.

**RESULTS:**

Two stable subtypes emerged: limbic‐predominant and hippocampal‐sparing. Limbic‐predominant participants were older, had higher amyloid burden, and faster memory decline, while hippocampal‐sparing individuals showed greater attention and executive function decline. Distinct thinning patterns were linked to specific network properties and gene expression profiles.

**DISCUSSION:**

These MRI‐based subtypes reflect underlying pathophysiological mechanisms and may aid in prognostication and clinical trial stratification.

**Highlights:**

- Two gray matter thickness subtypes can already be identified in preclinical stages, exhibiting distinct clinical characteristics and progression patterns.
- Individual subtype assignment remains stable over time.
- Longitudinal cortical thinning patterns follow distinct network‐ and transcriptomic‐based mechanisms within each subtype.

## 1 INTRODUCTION

Although traditionally viewed as a uniform sequence of biological events, Alzheimer's disease (AD) has been shown to exhibit high heterogeneity across various scales, with large variations in genetic, proteomic, and neuroanatomical individual profiles.^1^, ^2^, ^3^, ^4^ The diversity of neuropathological variants is already evident in aging individuals, likely contributing to variability in individual disease progression, and posing a major obstacle to achieving consistent clinical outcomes.^5^ As such, the effectiveness of tested disease‐modifying drugs may be compromised by the variability of pathological processes within the enrolled population. Although the presence of atrophy subtypes in the clinical stages of AD is now well established,^6^ the progression of these heterogeneous patterns from preclinical disease stages and the underlying mechanisms driving their development remain largely unclear. Understanding these processes is especially critical given recent efforts to advance early pharmacological interventions and population screenings.^7^

Previous evidence has converged on the identification of different putative AD atrophy subtypes.^8^, ^9^ A “typical‐AD” variant is often observed, showing neurodegeneration in hippocampal and association cortices. Partially overlapping with it, the “limbic‐predominant‐AD” pattern primarily involves the hippocampus and medial temporal cortices. Finally, the “hippocampal‐sparing AD” (or “diffuse”) variant is characterized by atrophy in associative cortices with sparing of the hippocampus.^6^ With the shift toward earlier primary and secondary prevention clinical trials, critical questions arise regarding the start of such heterogeneity and its stability along disease progression. Emerging evidence suggests the existence of atrophy subtypes already at prodromal stages,^2^ and other studies have shown the applicability of subtyping models in individuals with preserved cognition.^10^ In a recent work, the presence of two atrophy subtypes was observed in individuals enrolled in memory clinics.^11^ Interestingly, in participants who were considered atrophy‐negative (stage 0 of a certain subtype), baseline subtype attribution was predictive of longitudinal subtype conversion, suggesting that these individuals were already aligned with a specific trajectory even in the absence of overt atrophy. It is possible that such anatomical variants emerge during the lifespan and result in individual vulnerability for a specific disease or subtype. However, there is limited knowledge regarding the incidence and prevalence of atrophy subtypes in subjects without a dementia diagnosis.

**RESEARCH IN CONTEXT**

1. **Systematic review**: Distinct patterns of brain atrophy have been observed in Alzheimer's disease and its prodromal stages, suggesting the utility of magnetic resonance imaging (MRI) ‐based classification for identifying individual vulnerability before dementia. However, it remains unclear at what stage these signatures emerge, whether they remain stable over time, and how genetic and environmental factors contribute to their development and evolution.
2. **Interpretation**: Using data from two large multicenter cohort studies, our results propose a framework where latent anatomical changes emerge in older age and their patterns reflect underlying pathophysiological mechanisms and individual responses to aging‐related processes.
3. **Future directions**: MRI‐based thickness subtypes might inform patient stratification for prognostic purposes and patient selection in clinical trials.

A second crucial question relates to the mechanisms underlying these gray matter patterns and whether they are specific to each subtype. Recent multimodal imaging studies and imaging‐transcriptomic approaches^12^, ^13^ provide an efficient framework for bridging the multiscale organization of the brain and investigating biological determinants of atrophy propagation.^14^ Increasing evidence suggests that neurofibrillary tangles and subsequent AD‐related atrophy spread through connected brain regions in a prion‐like manner.^15^ Other studies suggest that regional transcriptomic vulnerability could be a strong determinant of atrophy progression.^14^ However, these studies have not considered disease heterogeneity. Possibly, different mechanisms drive cortical thinning within each subtype and could represent targets for specific treatments, providing a powerful tool for personalized medicine, and promoting individualized predictions of disease progression.

In this work, we aimed to answer these two fundamental questions by analyzing data from two large cohorts of older individuals with preserved cognition. First, we hypothesized that subtypes of gray matter regional vulnerability can be detected in these participants. Second, we examined the longitudinal evolution of the observed patterns of cortical thinning and hypothesized their propagation would be driven by distinct underlying mechanisms.

## 2 METHODS

**FIGURE 1:**
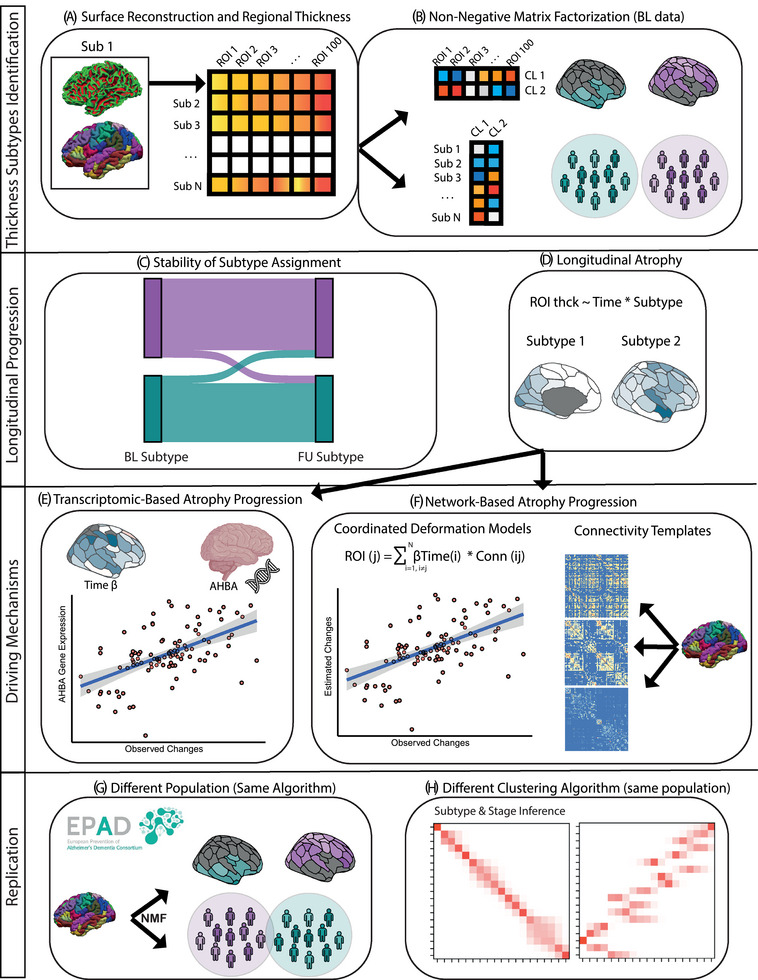
Schematic overview of methodology.

A schematic representation of the methods is shown in Figure 1.

### 2.1 Discovery cohort

We used data from the Amyloid Imaging to Prevent Alzheimer's Disease—prognostic and natural history study (AMYPAD–PNHS) consortium.^16^, ^17^ AMYPAD is a collaborative effort between multiple European cohorts, including participants above 50 years of age and with no diagnosis of dementia at baseline, based on a Clinical Dementia Rating (CDR) score < 1. Exclusion criteria were the presence of conditions associated with neurodegeneration or affecting cognition, or contraindication to MRI or positron emission tomography (PET). For this work, we selected participants with T1‐weighted (T1w) MRI and amyloid‐PET scans, resulting in a final sample of 1323 individuals. Of these, 662 participants had longitudinal magnetic resonance imaging (MRI) data available (follow‐up time = 3.66 ± 1.78 years, 573 had 2 visits, 89 had 3 visits).

### 2.2 Amyloid‐PET and other clinical data

Amyloid‐PET acquisition, processing, and harmonization in AMYPAD are described in previous works.^18^, ^19^ Briefly, PET scans were acquired 90–110 min post injection of 185 MBq (± 10%) of [^18^F]Flutemetamol or 350 MBq (± 20%) of [^18^F]Florbetaben, consisting of 4 frames of 5 min according to the standard protocol for each tracer.^20^, ^21^ An automated centralized pipeline (implemented by the IXICO clinical research organization) was used to process the PET scans. PET frames were co‐registered, averaged, and aligned to the corresponding MRI scan, which was parcellated using a subject‐specific multi‐atlas approach, that is, the learning embeddings for atlas propagation (LEAP) parcellation procedure.^22^ Standardized uptake value ratio (SUVr) images were obtained using LEAP parcellation masks using the whole cerebellum as a reference region in native space. SUVr values were transformed to Centiloids (CL) using the standard Global Alzheimer's Association Interactive Network (GAAIN) target region as a measure of global amyloid burden.^23^

*Apolipoprotein E (APOE)* genotype was determined following cohort‐specific procedures for blood sample analysis. *APOE*‐ε4 carriers were defined as having at least one ε4 allele. Cognitive outcomes for relevant cognitive domains were selected based on test availability or harmonization across AMYPAD parent cohorts. Distinct measures of immediate and delayed recall were used to assess performance in the memory domain, based on test availability across different parent cohorts. Digit Span Forward and Backward scores were used to assess performance in the attention (both), working memory (both), and executive functions (backward) domains. More details about cognitive tests and harmonization are given in the Supplementary Information.

### 2.3 MRI acquisition and processing

Details about MRI acquisition are given in previous publications.^24^ All included participants underwent three‐dimensional 3T‐T1w MRI on either a Philips (*n* = 854), Siemens (*n* = 455), or GE HealthCare (*n* = 14) scanner. T1w processing was performed with FreeSurfer v7.1.1 (https://surfer.nmr.mgh.harvard.edu/), including motion correction, skull stripping, intra‐subject template estimation (for longitudinal data only), brain parcellation, and estimation of regional gray matter thickness. The details of these procedures are described elsewhere.^25^, ^26^ We derived cortical thickness in 100 regions of interest (ROIs; Figure 1A) from the Schaefer atlas,^27^ from both baseline and follow‐up 3D T1w. Regions in the Schaefer atlas are annotated according to 7 canonical resting‐state networks, allowing for functional interpretation of the results. For replication purposes, cortical thickness values were also extracted for 64 regions of the Desikan Killiany (DK) atlas^28^.

### 2.4 Non‐negative matrix factorization

To identify clusters of participants based on MRI features, we used non‐negative matrix factorization (NMF) on baseline cortical thickness measures from the 100 ROIs (Figure 1B). NMF is a robust dimensionality reduction and clustering approach that has shown sensitivity in detecting clusters of atrophy (and other biomarkers) in previous studies.^1^, ^2^, ^29^ Given a number of features (regional cortical thickness) for each individual, NMF decomposes data into positive matrices and reduces the dimensionality of the features into fewer components that can be considered feature profiles or subtypes. Individuals can then be assigned to one subtype based on the similarity of their thickness values with the identified thickness subtypes. Following our hypothesis, we assigned all individuals (and all regions) to a subtype based on the highest NMF score. Raw thickness values (uncorrected for covariates) were used. Regional thickness values were first inverted so that higher values reflected decreased thickness, instead of preserved gray matter. To find the best number of fitting clusters, we used multiple fit statistics, ^30^, ^31^ including the cophenetic coefficient, silhouette coefficient, and change in residual sum of squares (see Supplementary Information for details).

### 2.5 Statistical analysis

#### 2.5.1 Subtypes characterization

We first examined differences between individuals assigned to different subtypes in demographics and other clinical characteristics. We used a logistic regression model to study differences between subtypes (dependent variable) in age, sex, global CL, and *APOE*‐ε4 carriership (independent variables), correcting for subtype assignment probability from NMF.

To explore subtypes’ longitudinal trajectories in clinical outcomes, we employed linear mixed models using longitudinal global CL or the scores in the selected cognitive tests as outcome variables. Predictors included participants' subtype assignment and its interaction with time. Models also included the correction for age, sex, global baseline CL, baseline NMF probability, and a random intercept on the participants. Within subtypes, we also studied the impact of having higher NMF scores on longitudinal outcomes (NMF score‐by‐time interaction). Higher NMF scores indicate observations that are more strongly associated with the assigned subtype, thus having a stronger contribution to the subtype profile.

#### 2.5.2 Subtype longitudinal stability

We then examined whether participants with longitudinal time points showed consistency in their cortical thickness subtype over time (Figure 1C). To achieve this, we selected the latest follow‐up T1w scan from each participant and assigned it to one of the baseline subtypes based on the correspondence (Pearson correlation) of cortical thickness patterns to the NMF subtypes. Participants were classified as “stable” or “unstable” based on whether the subtype remained the same across timepoints. Krippendorff's alpha coefficient was used to quantify subtype assignment agreement between baseline and follow‐up timepoints. Logistic regression models were used to study the impact of age, sex, follow‐up time, and baseline NMF probability (independent variables) on participants' subtype stability (binary dependent variable).

#### 2.5.3 Subtype‐specific longitudinal cortical thinning

We then investigated longitudinal cortical thinning within each subtype (Figure 1D). We used linear mixed effects regression models, including the effect of time (independent variable) on thickness values from each ROI. To study subtype‐specific mechanisms, we included a time‐by‐subtype interaction term and computed estimated marginal means within subtypes using the emmeans v1.11.2 package in R^32^. Models also included correction for age, sex, global CL, baseline NMF probability, and a random intercept on the participants.

#### 2.5.4 Network contribution to progression of cortical thinning

To assess whether longitudinal cortical thinning progresses preferentially following brain connectivity, we employed coordinated deformation models (Figure 1F).^14^ In this framework, network‐based changes of thinning within a region are estimated by multiplying the effect of time in connected regions (β from the linear model) by the strength of their connections to the region itself, resulting in an ROI‐wise map of connectivity estimated thickness changes. For each subtype, we built three coordinated deformation models using a functional, structural, and morphological similarity connectivity template, respectively. Details on the construction of connectivity templates are given in Supplementary Information. The resulting maps of estimated changes were then spatially correlated to the observed changes (β from the linear model), to find the connectivity template that best predicted the observed changes.

We used a two‐fold approach to evaluate the significance of spatial correlations. First, we ensured that the observed correlations were not driven by the topological characteristics of the connectivity templates (network/rewired null model). We generated 1000 random networks that preserved the degree sequence, connection weights distribution, the Euclidean distance between nodes, and the distance‐weight relationship, and recomputed the coordinated deformation model using these networks, resulting in a distribution of null spatial correlations. *Rewired p‐values* were then computed as the fraction of permutation values greater than the original spatial correlation value.

Second, we assessed whether the observed correlations were independent on the statistical autocorrelations typical of brain features distribution.^33^ To do so, we used BrainSMASH (https://brainsmash.readthedocs.io), a Python‐based package for statistical testing of spatially autocorrelated brain maps.^34^ Each subtype‐specific map of the effect of time on regional thickness was used as input to generate 1000 randomized brain maps while preserving the original spatial autocorrelation. We performed coordinated deformation models using these maps, resulting in a null distribution of spatial correlations. *Autocorrelations p‐values* were computed as the fraction of permutation values greater than the original spatial correlation value.

#### 2.5.5 Gene‐expression contribution to cortical thinning

To investigate whether gene expression plays a role in shaping the different patterns of cortical thinning progression (Figure 1E), we retrieved data from the open‐access Allen Human Brain Atlas (AHBA; http://human.brain‐map.org/), providing regional microarray expression data from six post‐mortem brains (one female, ages 24–57 years, 42.5 ± 13.38 years). Genes of interest were selected as being related to AD and other neurodegenerative processes from recent genome‐wide association studies (GWAS) for AD,^35^ white matter hyperintensities (WMH),^36^ cerebrovascular disease,^37^ limbic‐predominant age‐related TDP‐43 encephalopathy (LATE),^38^ posterior cortical atrophy (PCA)^39^ (see Supplementary Information and Table S1 for rationale and a full list of selected genes). For the selected 249 genes, we generated vectors storing gene expression for the 100 ROIs of the Schaefer atlas. AHBA data processing and extraction were performed with the open‐access abagen toolbox (https://github.com/rmarkello/abagen), using default parameters.^13^ We then performed spatial correlation analysis of the subtype‐specific effect of time (β of the linear mixed effects models) and each ROI‐wise gene expression map. Similarly, to the network analysis, we assessed the significance of the spatial correlations by controlling for statistical autocorrelations typical of brain features distribution.^33^ For each gene map, we computed 1000 null correlations with permuted β maps of the effect of time, and evaluated its significance as the fraction of permutation values greater than the original spatial correlation value.

Lastly, we performed gene over‐representation analysis (ORA) on the genes that showed significant correlation to longitudinal gray matter thinning within each subtype to evaluate enriched biological processes.^40^, ^41^, ^42^ We used the function enrichGO from the R package “clusterProfiler” ^43^ to perform ORA, with Gene Ontology^40^ as a reference gene source for functional profiling. ORA results were compared between subtypes using the compareCluster function from the same package and visualized through emapplot.

#### 2.5.6 Sensitivity analyses

To test the stability of our results, we replicated the main analyses across a variety of conditions. Specifically, the NMF algorithm and coordinated deformation models were repeated using a different parcellation, namely the DK atlas, to test for possible biases due to the functional properties intrinsic to the Schaeffer atlas. Moreover, the NMF algorithm was repeated only on a subset of participants who were amyloid positive, to test for subtype stability across disease stages, and on statistically harmonized thickness values, to assess robustness of the results regarding multi‐site data. NMF was also performed on age‐ and sex‐corrected thickness data to assess possible confounding effects. Moreover, the longitudinal models investigating immediate and recall memory scores differences between subtypes were repeated by iteratively taking out one cognitive test, to evaluate possible disproportional effects. All sensitivity analyses are described and reported in the Supplementary Information.

### 2.6 Replication

First, we aimed at replicating our clustering results on the same cohort using the Subtype and Stage Inference (SuStaIn) algorithm (Figure 1H). Contrary to NMF, SuStaIn simultaneously characterizes the heterogeneity and progression of the studied cross‐sectional biomarkers. A complete mathematical description of the SuStaIn algorithm is available elsewhere.^44^ More details about the SuStaIn application in this work are given in the Supplementary Information. Briefly, SuStaIn model fitting consists of an iterative procedure that simultaneously estimates subtype event sequences and subtype classification for a preselected number of subtypes. Average cortical thickness was *z*‐standardized against a reference group of cognitively unimpaired (CDR = 0) and amyloid‐negative participants on amyloid‐PET visual inspection (*N* = 653). A threshold of −1 *z*‐scores was chosen as the event threshold, consistent with earlier work.^44^ Independently from the NMF analysis, the optimal number of subtypes was selected using ten‐fold cross‐validation, with the out‐of‐sample likelihood used to compute the cross‐validation information criterion (CVIC). The model with the lowest CVIC was deemed optimal. Additionally, to assess the consistency of subtype assignment, SuStaIn modeling was repeated using a threshold of −1.96 *z*‐scores, which represents the most commonly used threshold for statistical abnormality.

Subsequently, we aimed at replicating our clustering results by using NMF on a different cohort (Figure 1G). To this end, we retrieved data from the European Prevention of Alzheimer's Dementia (EPAD) multicenter study.^45^ For our replication, we only used EPAD participants who were not subsequently included in AMYPAD, resulting in a replication sample of 927 subjects. Demographics and clinical characteristics are reported in Table S2. NMF cluster number optimization and fitting were performed in the same way as for the discovery cohort.

## 3 RESULTS

### 3.1 Discovery cohort characteristics

Cohort characteristics, stratified by CDR score, are presented in Table 1. The mean age was 68 (± 8.7) years, 571 (43.2%) were men, and 236 (17.8%) had a global CDR score of 0.5. The average Mini‐Mental State Examination (MMSE) score was 28.8 (± 1.49), with lower scores in subjects with CDR = 0.5. At least one *APOE* ε4 allele was present in 40% of participants. In the subset with longitudinal data available, the average follow‐up time was 3.66 years (± 1.78), 573 participants had 2 MRI visits, and 89 had 3 MRI visits.

**TABLE 1:** Cohort characteristics.

Parameter	Overall (*N* = 1323)	CDR = 0 (*N* = 1044)	CDR = 0.5 (*N* = 236)
Age, years. mean (SD)	68.00 (8.65)	67.23 (8.78)	71.44 (7.53)
Sex, male. *N* (%)	571 (43.2)	433 (41.5)	126 (53.4)
MMSE, score, mean (SD)	28.84 (1.49)	29.15 (1.03)	27.55 (2.31)
Education, years, mean (SD)	14.59 (3.93)	14.60 (3.99)	14.53 (3.80)
Amyloid PET, Centiloid, mean (SD)	18.84 (31.50)	14.28 (26.14)	37.60 (42.18)
Amyloid PET, visual classification positive, *N*(%)	299 (22.7)	182 (17.5)	103 (44.2)
APOE ε4, carrier, *N* (%)	530 (40.1)	398 (38.1)	117 (49.6)
Follow‐up time (*N* = 662), days, mean (SD)	668.49 (810.90)	812.16 (823.28)	148.83 (501.38)
No. of visits, *N* (%)			
1	661 (50%)	417 (39.9%)	204 (86.4%)
2	573 (43.3%)	538 (51.5)	32 (13.6%)
3	89 (6.7%)	89 (8.5%)	0 (0%)

Abbreviations: *APOE, apolipoprotein E;* CDR, Clinical Dementia Rating; MMSE, Mini‐Mental State Examination; PET, positron emission tomography; SD, standard deviation.

### 3.2 Subtype definition

Two subtypes showed the optimal NMF fit according to goodness‐of‐fit metrics (Table S3). Sensitivity analysis with different numbers of subtypes is reported in the Supplementary Information and Figure S1.

**FIGURE 2:**
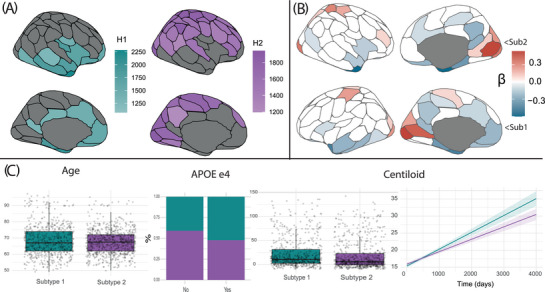
Thickness Subtypes, demographics, and clinical differences. (A) The regional NMF scores (H) for regions assigned to each identified gray matter thickness subtype. Regional assignment to subtypes was based on NMF probability. (B) Significant (*p* < 0.05) regional differences between the two subtypes. Differences are reported as standardized beta values of linear models predicting regional thickness values using subtype assignment. (C) Differences in age, *APOE* ε4 carriership, baseline, and longitudinal amyloid (model estimates). *APOE, apolipoprotein E;* NMF, non‐negative matrix factorization.

Regional thickness subtypes are shown in Figure 2A. 35 ROIs were assigned to the first subtype based on NMF probability. These were mostly located in medial areas (e.g., cingulate, precuneus), medial‐temporal, and lateral‐temporal areas. Functional annotations (from the correspondence of Schaefer 100 atlas regions to canonical resting state networks)^27^ showed a large involvement of regions implicated in the default mode network, and also of the limbic, control, and ventral attention networks. In total, 601 participants were assigned to this subtype (mean NMF probability = 0.62 ± 0.08). Of these, 442 (73.5%) were amyloid negative on visual read, while 159 (26.5%) were amyloid positive. 65 ROIs were assigned to the second subtype based on NMF probability, mostly located in the occipital, lateral parietal, and lateral frontal regions. Compared to the first subtype, functional annotations showed larger involvement of the dorsal attention, control, and visual networks in this subtype. In total, 722 participants were assigned to the second subtype (mean NMF probability = 0.60 ± 0.06). Of these, 579 (80.2%) were amyloid negative on visual read, while 143 (19.8%) were amyloid positive. Regional effects of subtype assignment on gray matter thickness (*p* < 0.05) are shown in Figure 2B. Highly comparable subtypes and results were found across all performed sensitivity analyses (Figure S2–S5).

### 3.3 Subtypes characterization

**FIGURE 3:**
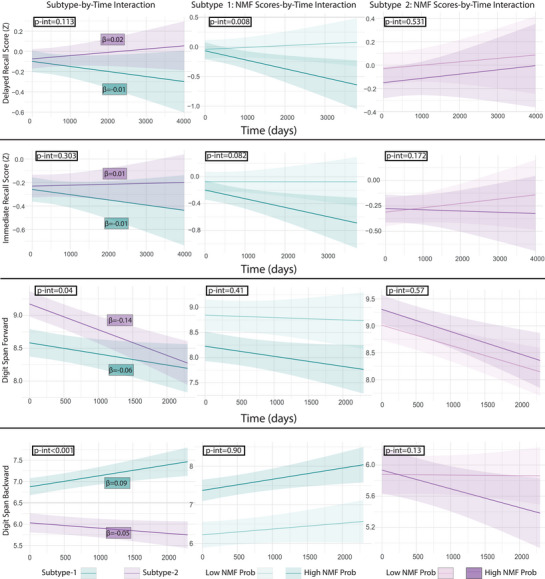
Cognitive performance and decline in thickness subtypes. The left column shows time‐by‐subtype estimates in the four assessed cognitive domains. *p*‐Values of the interactions are reported in black. Beta values report the association with cognitive performance within each subtype. The middle and right columns show the association of NMF probability with longitudinal (interaction with time) cognitive outcomes. Models used continuous NMF probability; median split was used to create groups for visualization purposes. NMF, non‐negative matrix factorization.

All coefficients of models investigating differences between subtypes are reported in Tables S4, S5, and S6. Participants classified as subtype 1 were older, had higher amyloid CL values, and were more often *APOE* ε4 carriers (Figure 2B). When looking at longitudinal outcomes, both subtypes had significant amyloid accumulation over time (subtype1: *β* = 1.80, *p* < 0.001; subtype2: *β* = 1.33, *p* < 0.001), with the first subtype showing faster accumulation rates (*p*‐interaction = 0.007; Figure 2B). Higher NMF scores were related to faster amyloid accumulation in both subtypes (subtype1: *p*‐interaction = 0.012; subtype2: *p*‐interaction = 0.006). Subtype differences in cognitive scores progression are shown in Figure 3. Performance in memory cognitive tests (both delayed and immediate recall) was not found to deteriorate differently between subtypes. However, within subtype 1 participants, higher NMF scores were related to faster decline in delayed memory performance (*p*‐interaction = 0.008). In the Digit Span Forward test, subtype 2 had better scores at baseline, but declined faster compared to subtype 1 (*p*‐interaction = 0.04). In the Digit Span Backward, subtype 2 had worse scores at baseline and declined faster (*p*‐interaction < 0.001). Sensitivity analyses demonstrated consistent effects across distinct memory tests (Table S7).

### 3.4 Subtype stability over time

Out of 662 participants with longitudinal data, high agreement between baseline and longitudinal subtype assignment was observed (Krippendorff's alpha = 0.79). Only 68 (9.7%)—34 from each subtype— showed inconsistent subtype assignment and thus were classified as “unstable”. Logistic regression showed that lower NMF probability was related to subtype instability (OR = 1.027, confidence interval [CI] = 1.020–1.036; *p* < 0.001), while age, sex, and follow‐up time did not show significant associations. All coefficients are reported in Table S8.

### 3.5 Determinants of cortical thinning progression

#### 3.5.1 Subtype‐specific longitudinal thinning patterns

**FIGURE 4:**
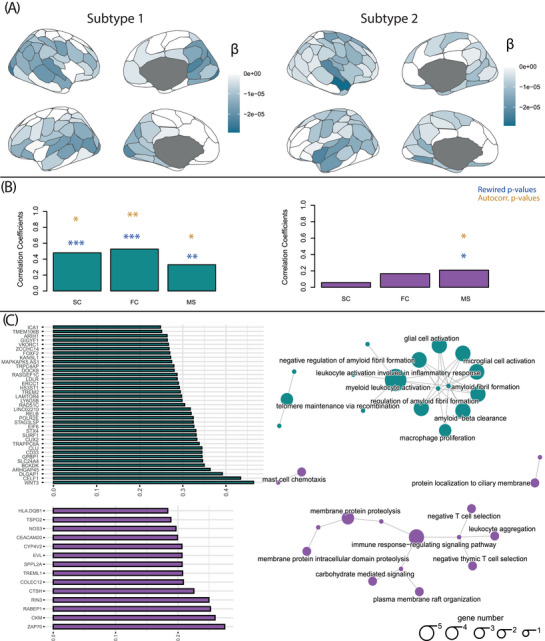
Longitudinal cortical thinning and driving mechanism within thickness subtypes. (A) The effect of time on regional gray matter thickness values within the two subtypes. (B) The results of the coordinated deformation models, reporting significance based on the two computed *p*‐values for each connectivity template, within each subtype. (C) The results of the imaging transcriptomic analysis. On the left, the genes that showed a significant correlation of their expression maps with longitudinal cortical thinning within each subtype. On the right, a visualization of the enriched pathways within the significant genes. FC, functional connectivity; MS, morphological similarity; SC, structural connectivity.

Linear mixed‐effect models revealed widespread significant effects of the time‐by‐subtype interaction term. Figure 4A shows the effect of time on thickness within each subtype (estimated marginal means of linear trends). *p*‐Values are reported in Table S9. Subtype 1 showed significant reductions of thickness over time in lateral superior temporal regions and posterior medial regions, while Subtype 2 had significant reductions of thickness over time mostly in dorsal regions, including dorsal parietal and frontal regions, and also in lateral temporal regions. Similar longitudinal thinning patterns were observed when repeating the analysis using a different cortical parcellation (Figure S2).

#### 3.5.2 Network contribution to cortical thinning

Figure 4B shows the results of the coordinated deformation models. Longitudinal cortical thinning progression within subtype 1 was predicted by all connectivity templates, and most strongly by functional connectivity. Within subtype 2, instead, morphological similarity was the only significant predictor of longitudinal thinning. Results were consistent with both null models used (rewired and autocorrelation *p*‐values). Similar results were observed when repeating the analysis with a different cortical parcellation (Figure S2). Interestingly, structural connectivity yielded stronger predictive power than functional connectivity when an anatomically defined atlas was used, suggesting that intrinsic properties of the chosen parcellation may partly account for these findings.

#### 3.5.3 Gene‐expression contribution to cortical thinning

The results of the imaging transcriptomic analysis are shown in Figure 4C. Subtype 1 longitudinal patterns of cortical thinning were found to correlate with 37 of the investigated genes, of which 25 stemmed from the AD and related dementias GWAS, two from the WMH GWAS, eight from the cerebrovascular GWAS, one from the LATE GWAS, and two from the PCA GWAS. When performing ORA, these genes were enriched for several biological processes, including regulation of amyloid formation and clearance, neuroinflammatory responses, and lipid metabolism. Longitudinal patterns of cortical thinning of subtype 2 were correlated with 11 of the investigated genes, 7 of which were from the AD GWAS, 2 from the WMH, 1 from the cerebrovascular, and 1 from the PCA GWAS. When performing the ORA, these genes were enriched for biological pathways related to immune activation, immune system processes, and T‐cell regulation.

### 3.6 Replication

Fit coefficients of NMF sensitivity analyses are reported in Tables S10–S12.

**FIGURE 5:**
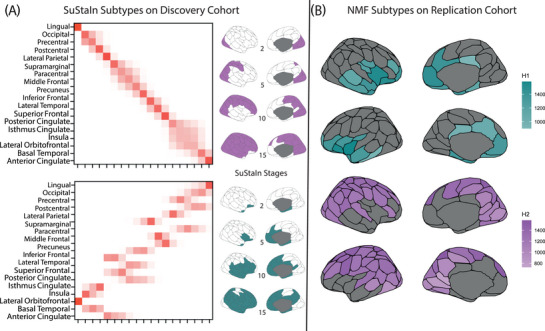
Replication. (A) The subtypes identified using the SuStaIn algorithm on the discovery cohort. Rows represent regions of interest and columns represent stages. A visualization of the brain of the observed subtypes and stages is reported. Panel B shows the results of the NMF algorithm on the replication cohort. NMF, non‐negative matrix factorization.

When using SuStaIn, the optimal number of clusters was two, in line with our main results (Figure S6). Different from NMF, SuStaIn also provides a temporal ordering of the cross‐sectional biomarkers. The two identified subtypes showed large overlap with our NMF subtypes (Figure 5A). The first one had initial thinning in occipital areas, followed by dorso‐parietal, and eventually frontal areas, similar to what is observed in our NMF subtype 2. When only looking at participants with stage > 0, of the 344 participants assigned to this subtype, 93.6% (322) had been assigned to our NMF subtype 2 in the discovery analysis. In the second SuStaIn subtype, cortical thinning progressed from medial temporal to medial frontal and lateral temporal regions, with a pattern similar to the NMF subtype 1. When only looking at participants with stage > 0, of the 307 participants assigned to this subtype, 96.4% (296) had been assigned to our NMF subtype 1 in the discovery analysis. When replicating SuStaIn modeling with a threshold of 1.96 z‐scores, the optimal number of subtypes and thinning patterns were confirmed (see Supplementary Information and Figure S7 for details).

We then replicated our clustering results on a different cohort using the same algorithm. EPAD (replication) cohort characteristics are shown in Table S2. When NMF was run on EPAD, the optimal number of clusters was again 2 (Table S13). The two identified subtypes largely overlapping with the ones from our discovery cohort and are shown in Figure 5B. Twenty‐nine regions were assigned to the first subtype, mostly in limbic, frontal, and temporal areas. Of these, 28 (96.6%) had been assigned to subtype 1 in our discovery cohort. Sixty‐four regions were assigned to the second subtype, mostly in occipital, lateral parietal, and frontal areas. Of these, 64 (97.0%) had been assigned to subtype 2 in our discovery cohort.

## 4 DISCUSSION

Using data from two large cohorts of individuals without a diagnosis of dementia, we identified two consistent cortical thickness subtypes using different classification algorithms and investigated their clinical differences, longitudinal trajectories, and the mechanisms underlying their differential progression. Our findings reinforce existing literature on the presence of regional gray matter vulnerability signatures and reveal their emergence already in participants without overt pathological changes. We demonstrate that these subtypes were related to distinct clinical characteristics and that the participants’ subtype assignments remain stable over time. We found subtype‐specific longitudinal cortical thinning patterns and showed that unique network‐based and transcriptomic factors drive thinning propagation in each subtype.

Although our cohort did not include individuals with a clinical diagnosis of dementia, the two subtypes identified in this large asymptomatic population align with previous investigations of atrophy patterns in prodromal and clinical AD.^6^ A limbic‐predominant subtype, corresponding to our NMF subtype 1, and a hippocampal‐sparing subtype, corresponding to our NMF subtype 2, have consistently been identified across MCI,^11^ prodromal AD,^2^ and clinical AD stages using various imaging modalities. Less evidence exists on the presence of these subtypes in the normal aging population. In one study of cognitively unimpaired individuals, subtypes were identified as cortical‐ and hippocampal‐predominant atrophy, with the latter being more prone to cognitive decline and at higher risk of developing AD in the future.^46^ The clinical relevance of atrophy subtype characterization in individuals without dementia was further confirmed over a 4‐year follow‐up in a different cohort.^47^ In another recent work using two independent memory clinic‐based cohorts,^11^ two atrophy subtypes, strongly similar to the one we found, showed distinct clinical characteristics and high stability over longitudinal time points. In all those studies, individuals with atrophy in limbic and medial‐temporal regions (limbic‐predominant) had more evidence of AD pathology and faster decline in several cognitive domains, mostly including memory capabilities. However, hippocampal‐sparing variants also showed abnormal AD biomarkers and cognitive decline in different domains.^48^

Consistent with the dimensional conceptualization of brain atrophy patterns^6^ and neuroimaging phenotypes as a tool to capture heterogeneity,^49^ our findings reveal that even participants without any disease can have AD‐like signatures of cortical thinning, suggesting selective vulnerability, which may predispose toward individual risk of progression to a disease type. In this view, population studies of aging individuals offer valuable insights into the heterogeneity of the brain aging process in the absence of specific diseases.^50^, ^51^ Together with recent literature,^52^ our findings propose a framework where anatomical changes can already be measured in the general population as latent manifestations linked to underlying vulnerability. This heterogeneity may stem from a combination of multiple sources encompassing genetics, environmental, and other modifiable risk factors, such as vascular health, lifestyle, and comorbidities. Given the need for earlier interventions, these MRI‐based general dimensions of variability might provide critical insights in the context of memory clinics and brain health services,^7^ supporting population screening and individual risk stratification, as well as providing data‐informed guidelines for patient selection in clinical trials. Specifically, understanding patient‐specific regional patterns of gray matter vulnerability and assigning individuals to distinct subtypes could facilitate the creation of more homogeneous therapeutic target groups. This, in turn, would enable the development of individualized interventions tailored to the predominant underlying pathological mechanisms—such as amyloid deposition, vascular dysfunction, or neuroinflammation.

The existence of such anatomical patterns and their atrophy progression might, in fact, be driven by differential molecular mechanisms, genetic predispositions, or network vulnerabilities, posing important challenges for accurate intervention. Few studies have investigated mechanisms underlying subtype‐specific progression. Using connectivity and transcriptomic group‐level information, we characterized the mechanisms driving cortical thinning progression that differentiate the two subtypes. The first identified subtype (limbic‐predominant) had longitudinal thinning patterns that were predicted by several types of connectivity matrices. The notion that AD pathology and atrophy progress predominantly along neural networks is well‐established in the field and suggests prion‐like mechanisms underlying AD pathology spreading.^15^, ^53^ Several studies have shown that tau and atrophy propagation can be modeled using functional connectivity, also at an individual level.^54^ Similar results have been observed when using structural connectivity measures.^55^, ^56^ Less evidence exists about the role of regional morphological similarity networks. The typical AD pattern of neurodegeneration may therefore be explained by the vulnerability of highly interconnected hub regions, which are disproportionately impacted due to their central role in network connectivity and their high metabolic activity, rendering them more susceptible to activity‐dependent pathological processes.^57^ Interestingly, the finding that the choice of parcellation biases the results toward one connectivity type or the other supports a model in which atrophy in this subtype progresses along general brain connectivity principles, which can be partially captured by different MRI modalities. Contrary to the first, the second subtype —characterized by diffuse atrophy—had longitudinal degeneration only predicted by morphological similarity patterns.^58^ Macroscale similarity networks encompass information about cytoarchitectonic and myeloarchitectonic similarity between regions at the microscale, for example, related by their lamination and myelination.^59^ Individuals in this subtype might therefore show atrophy related to the intrinsic structural properties shared across regions with similar cytoarchitectonic and myeloarchitectonic features, potentially reflecting vulnerability driven by developmental or evolutionary constraints rather than activity‐dependent processes or network hub dynamics.^60^

This distinct regional vulnerability to cortical thinning progression may further be explained by differences in transcriptomic and cellular vulnerability. The first subtype's longitudinal patterns of atrophy correlated with spatial patterns of gene expression linked to amyloid formation, clearance, and microglial cell activation, which have been widely described in AD literature. This observation further underscores the possibility of identifying meaningful MRI‐based subtypes in the context of brain health clinics, which could be relevant for assessing individual risk of disease progression, and guiding personalized pharmacological interventions. pharmacological interventions. Together with the network analysis, this finding suggests the existence of a regional vulnerability gradient to AD‐specific processes based on regional gene expression levels and connectivity profiles. The second subtype had longitudinal degeneration spatially correlated with genes involved in immune and T‐cell activation pathways. These mechanisms have increasingly been considered central regulators of neurodegenerative diseases,^61^, ^62^ as a maladaptive response to brain damage due to aging. The specific involvement of neuroinflammatory processes and immune responses in driving the progression of this atrophy subtype may be due to regional differences in brain‐resident (for example, microglia) or infiltrating immune cells (for example, T cells) distribution.^63^, ^64^ These factors can, for example, impair blood–brain barrier permeability and induce neuroinflammation as a response to cerebrovascular damage.^65^, ^66^ These findings offer insights into the heterogeneity of biological pathways underlying neurodegeneration and suggest that multimodal interventions and risk modification may be necessary for individuals presenting non‐typical patterns.

Collectively, our results extend previous literature and depict a framework where MRI‐based measures can capture very early individual regional vulnerability patterns that remain stable in time. These signatures encompass crucial insights into pathophysiological mechanisms and individual responses to aging‐related processes and could represent important outcomes for early and personalized intervention in memory clinics. The observed patterns and underlying pathophysiological profiles could thus be considered brain traits, or ageotypes,^67^ representing individual vulnerability to certain risk factors, providing important tools for accurate disease stratification and forecasting.

Some limitations should be considered. Unlike in previous works, we did not use any control group to compare atrophy in our subtypes. NMF algorithms assign everyone to a subtype based on the similarity of the pattern of observed values. However, this is within the scope of this manuscript, as we aimed to demonstrate that regional vulnerability would already be measurable and informative in the absence of overt atrophy. The so‐called atrophy subtypes could thus be considered regional susceptibility patterns that differentiate across individuals throughout the aging process. Another limitation concerns the investigation of mechanisms driving atrophy. In this context, the performed analyses were used to describe population‐level trends and group effects. These approaches do not allow for modeling pathological progression at the individual level. At the same time, our findings provide important insights into the pathophysiological nature of disease heterogeneity and might be of interest for clinical applications, such as the identification of at‐risk individuals and optimization of intervention strategies, as well as stratification for enrollment in clinical trials. Future studies could employ more advanced statistical methods to characterize population‐level longitudinal patterns, such as incorporating individualized slopes or modeling non‐linear trajectories. Future studies should also evaluate differences between subtypes in markers of cerebrovascular damage, such as WMH, microbleeds, and enlarged perivascular spaces, in order to better elucidate the contribution of vascular and inflammatory processes to subtype‐specific patterns of atrophy. Finally, the cognitive domains used to compare performance between the two subtypes, and specifically episodic memory scores, were based on heterogeneous tests performed in each sub‐cohort.

## CONFLICTS OF INTEREST STATEMENT

F.B. is supported by Engineering and Physical Sciences Research Council (EPSRC), EUJU (IMI), National Institute for Health and Care Research—Biomedical Research Center (NIHR‐BRC), General Eletronic (GE) HealthCare; he is a consultant for Combinostics, IXICO, and Roche; participates on advisory boards of Biogen, Prothena, and Merck; and is a co‐founder of Queen Square Analytics. L.E.C. has received research support and speakers fee from GE HealthCare Ltd. and Springer Healthcare (paid to institution). M.P. #NEXTGENERATIONEU (NGEU) and funded by the Italian Ministry of University and Research (MUR), National Recovery and Resilience Plan (NRRP), project MNESYS (PE0000006) – A Multiscale integrated approach to the study of the nervous system in health and disease (DN. 1553 11.10.2022). T.G.O. has been a consultant for Sonae, Guidepoint and Lilly, has received fees as a speaker from Eisai and conference fees covered from Roche. N.P.O. is a consultant for Queen Square Analytics Limited (UK) on unrelated topics. M.B. has consulted for Grifols, Araclon Biotech, Roche, Biogen, Lilly, Merck, Novo‐Nordisk; has served in the Advisory Boards from Grifols, Roche, Lilly, Araclon Biotech, Merck, Biogen, Novo‐Nordisk, Bioiberica, Eisai, Servier, Schwabe Pharma; received fees from lectures from Roche, Biogen, Grifols, Nutricia, Araclon Biotech, Novo‐Nordisk, Eisai, Terumo, Schwabe Pharma; and reports research funding from Life Molecular Imaging, Bioiberica, Grifols, Araclon Biotech, Lilly, Roche, Janssen, Alzehon, Cortyzime, Novo Nordisk, Schwabe Pharma. M.M. has consulted for F. Hoffmann‐La Roche Ltd. and has served in the Spanish Scientific Advisory Board for biomarkers of Araclon Biotech. G.S. has received speaker fees from Springer and Adium. L.L., M.T., L.P., F.M., M.K., G.P., E.S.L., A.M.W., H.J.M.M., D.A., A.B., C.B., C.B., G.F., W.F., G.B.F., R.G., J.D.G., B.J.H., F.J., A.M., C.R., M.S., M.S., A.W.S., B.M.T., D.V.G., R.V., P.J.V., and L.R. have nothing to disclose. Author disclosures are available in the Supporting Information

## CONSENT STATEMENT

All EPAD participants provided written informed consent.

## Supporting information



Supporting Information

Supporting Information

Supporting Information

Supporting Information
