# Supplementary material for: Cortical thickness subtypes in cognitively unimpaired individuals: Differential network and transcriptomic vulnerability to cortical thinning
Source: Alzheimers Dement. 2025 Oct 14;21(10):e70762. doi: 10.1002/alz.70762 (PMC12519513; doi:10.1002/alz.70762)
Supplement: Supplementary file 1 — Supporting Information [file ALZ-21-e70762-s002.docx]

Supplementary Materials

# Supplementary Methods

## Neuropsychological Evaluation

The AMYPAD PNHS data collection is a combination of prospective and historical data from 32 European sites across 10 different countries: Belgium, France, Germany, Netherlands, Spain, Sweden, Switzerland, United Kingdom, Italy, and Greece. These sites provide their data through 10 Parent Cohorts (PC). Although following standard procedures, neuropsychological assessment was performed with cohort- and site-specific procedures and using partially different tests. In this study, we chose to restrict our analysis to neuropsychological evaluations related to memory and attention cognitive domains. Several reasons for this choice were considered:

1. Memory and attention related cognitive functions are often observed to be among the first to decline in pathological ageing[(Murman 2015)](https://paperpile.com/c/jYy4u9/oLpCL)
2. As such, the assessment of these cognitive functions is often performed in memory clinics and population studies of aging individuals. Indeed, most cohorts performed thorough memory, attention and executive function examinations, thus providing a wider availability of data
3. In AMYPAD, all cohorts performed the digit-span test (for the attention domain) allowing us to use this harmonized information

For these reasons, we decided to use 2 measures of memory performance (delayed and immediate) and 2 measures related to attention and executive control (digit span backward and forward).

For the delayed recall memory domain, 5 different test versions were used across cohorts. Of the 1197 participants included in this analysis, 395 were evaluated using the RBANS delayed recall score[(Yang et al. 2009)](https://paperpile.com/c/jYy4u9/Hcskf), 245 with the RAVLT delayed recall score[(Lezak 1998)](https://paperpile.com/c/jYy4u9/bTBOD), 49 with the CERAD recall score[(Morris et al. 1989)](https://paperpile.com/c/jYy4u9/HsvCn), 299 with the FCSRT delayed free recall score[(Grober and Buschke 1987)](https://paperpile.com/c/jYy4u9/2WTgF), and 209 with the WMS delayed recall score.

Similarly, for the immediate recall memory domain, 5 different test versions were used across cohorts. Of the 1197 participants included in this analysis, 395 were evaluated using the RBANS list learning score[(Yang et al. 2009)](https://paperpile.com/c/jYy4u9/Hcskf), 245 with the RAVLT total immediate recall score[(Lezak 1998)](https://paperpile.com/c/jYy4u9/bTBOD), 49 with the CERAD total recall score [(Morris et al. 1989)](https://paperpile.com/c/jYy4u9/HsvCn), 299 with the FCSRT immediate free recall score[(Grober and Buschke 1987)](https://paperpile.com/c/jYy4u9/2WTgF), and 209 with the WMS total immediate recall score[(Alegret et al. 2013)](https://paperpile.com/c/jYy4u9/t4hdV). Following previous works on the same cohort, memory cognitive scores were Z-scaled within each cohort and integrated across cohorts.

## Non-Negative Matrix Factorization

Non-negative matrix factorization (NMF) was performed in R (version 4.3.1). Clustering of grey matter thickness values across regions of interest was performed with the R package Nonnegative Matrix Factorization (NMF).[(Gaujoux and Seoighe 2010)](https://paperpile.com/c/jYy4u9/KwhjA) We performed NMF using the non-smooth NMF algorithm that enhances the sparsity of the cluster solution.[(Pascual-Montano et al. 2006)](https://paperpile.com/c/jYy4u9/MFTPm).

In both the discovery and replication dataset, we determined the optimal number of clusters by assessing the cophenetic coefficient, silhouette coefficient, and change in residual sum of squares. The cophenetic coefficient represents the stability of the individual cluster assignment and varies from 0 to 1, with highest values (>0.9) showing high stability of the cluster solution across iterations. The silhouette coefficient represents the level of separation between clusters, with values above 0.5 usually considered to be acceptable. The residual sum of squares (RSS) represents how much of variation remains unexplained in the model fit. Its changes from a lower cluster solution provide information of the improvement in the fit, and should be bigger in the observed data compared to a randomized solution. For two to six cluster solutions, estimates of the cophenetic and silhouette coefficients were obtained with 50 repeats of the non-smooth NMF algorithm in the original and random data. The Residual Sum of Squares (RSS) was computed for each clustering solution by applying NMF to both the original data matrix and a randomized version. The change in RSS was calculated as the difference in RSS between consecutive cluster solutions. The RSS change for the two-cluster solution was determined by comparing its RSS to that of an NMF fit with a single cluster. After determining the optimal number of clusters in each dataset, NMF was run for 500 repeat runs.

## Connectivity Templates

To perform coordinated deformation models we employed a functional, structural and a morphological similarity connectivity template providing information about normative connectivity between regions of interest. To build these connectivity templates, we used a selection of participants from the EPAD cohort that were not subsequently included in AMYPAD (similarly to what was done for the replication cohort). Specifically, we included participants with no evidence of AD pathology (A-T-) and with no cognitive impairment (CDR = 0), resulting in a sample of 456 selected EPAD participants. Using these participants ensured to build normative connectomes that represent the average connectivity of healthy individuals within the same range of age of the studied population. We used participants’ T1w, fMRI and DWI scans to build individuals’ connectomes. Briefly, T1w were pre-processed using FreeSurfer 7.1.1 and fed into the Morphometric INverse Divergence (MIND)[(Sebenius et al. 2022)](https://paperpile.com/c/jYy4u9/WErsz) that estimates regional similarity based on multivariate distributions of several morphological features. FMRI scans were processed using fmriprep v23.0 [(Esteban et al. 2019)](https://paperpile.com/c/jYy4u9/mvBFE). Regional time series were correlated to obtain individual functional connectomes. DWI scans were pre-processed using qsiprep v0.19. Probabilistic tractography was performed following standard MRTrix procedures. Structural connectomes were built using SIFT2 algorithm[(Smith et al. 2015)](https://paperpile.com/c/jYy4u9/LnoNA).

Individuals’ connectomes per each modality were averaged and proportionally thresholded at 30% to create connectivity templates.

## Candidate Genes Selection

For the imaging-transcriptomic analysis, we selected relevant genes to compute their expression profiles across the 100 regions of interest. Gene selection was based on recent literature on genetic determinants of age-related and neurodegenerative disorders[(Balusu et al. 2023)](https://paperpile.com/c/jYy4u9/EaxiZ). Candidate genes were selected when being annotated in a recent relevant genome wide association study (GWAS). We selected recent GWAS for AD,[(Bellenguez et al. 2022)](https://paperpile.com/c/jYy4u9/l0Fl1) white matter hyperintensities,[(Persyn et al. 2020)](https://paperpile.com/c/jYy4u9/fouWe) cerebrovascular disease,[(Mishra et al. 2022)](https://paperpile.com/c/jYy4u9/nnCZh) limbic-predominant age-related TDP-43 encephalopathy (LATE),[(Nelson et al. 2024)](https://paperpile.com/c/jYy4u9/IuGvV) and posterior cortical atrophy (PCA).[(Schott et al. 2016)](https://paperpile.com/c/jYy4u9/lo95z) Supplementary Table 1 reports the selected genes, the source GWAS and, if present, the overlap with other selected GWAS.

## Subtype and Stage Inference (SuStaIn)

To replicate our NMF clustering results with a different and widely used[(Baumeister et al. 2024; Vogel et al. 2021; Collij et al. 2022)](https://paperpile.com/c/jYy4u9/Hn516+hPMMA+wSPM6) method we decided to employ the Subtype and Stage Inference (SuStaIn) algorithm.[(Young et al. 2018)](https://paperpile.com/c/jYy4u9/uwQrI) Differently from NMF, SuStaIn simultaneously subtypes in both space and time providing *progression* clusters each having a unique spatiotemporal signature of abnormality accumulation. SuStaIn provides probabilistic subtype and stage assignment. While we examined a cohort with relatively limited coverage of the disease spectrum, SuStaIn is still important to confirm the presence of the NMF subtypes and assure that these are not an expression of time-dependent (stage-dependent) disease presentations. For computational reasons we reduced the dimensionality of the biomarkers: Schaefer ROIs were assigned to corresponding Desikan-Killiany (DK) ROIs,[(Desikan et al. 2006)](https://paperpile.com/c/jYy4u9/quT9) by assigning each Schaefer ROI to the closest DK ROI based on euclidean distance. Schaefer ROIs assigned to the same DK ROI were averaged. Subsequently, as previously described,[(Collij et al. 2022)](https://paperpile.com/c/jYy4u9/wSPM6) DK ROIs were assigned to macroregions that were further averaged across hemispheres to obtain the following 19 regions of interest: precentral gyrus; postcentral gyrus; paracentral lobule; medial and lateral orbitofrontal gyri; lateral parietal lobe (superior and inferior parietal lobules); precuneus; anterior, posterior, and isthmus cingulate gyri; supramarginal gyrus; insula; inferior, middle, and superior frontal gyri; lingual gyrus; occipital lobe (cuneus, lateral occipital gyrus and pericalcarine cortex); lateral temporal lobe (middle, transverse, and superior temporal gyri, superior temporal sulcus, and temporal pole); basal temporal lobe (parahippocampal, fusiform and inferior temporal gyri, and entorhinal cortex). For comparison with NMF results and visualization, the derived subtypes and stages were mapped back to Schaefer ROIs.

## NMF vs SuStaIn

In our study, we decided to use NMF as a primary algorithm and SuStaIn for replication purposes. The choice of NMF was mostly based on three considerations:

1. Previous works, including ones from our group[8](https://paperpile.com/c/l2wPLe/mYCS), have successfully used NMF to identify disease subtypes using cortical gray-matter volume and thickness data. While this is also true for SuStaIn, these results confirm that NMF can successfully be used for this purpose.
2. As highlighted in many instances in the main text, the aim of our work was to find latent dimensions of regional vulnerability, that are informative of underlying pathophysiology and disease progression in older individuals. In this view, the concept of stage (inherent in SuStaIn) was less relevant. While on one side stages can represent a pseudo-pathological progression, for the specific aim of this study the combination of NMF + longitudinal data seemed more appropriate.
3. The third reason concerns the characteristics of the investigated cohort. Compared to other SuStaIn-based studies, the cohort is composed of a more homogeneous population in terms of disease course coverage. In fact, all included participants don't have cognitive impairments and might only present initial AD pathology. Using SuStaIn as the main clustering algorithm, as opposed to NMF, could therefore force the assumption of the existence of stages in a population with limited disease span.

## Replication Cohort

As described in the main text, to replicate NMF results we retrieved data from the European Prevention of Alzheimer’s Dementia (EPAD) multicenter study.[(Ritchie et al. 2020)](https://paperpile.com/c/jYy4u9/CJC2n) We only used EPAD participants that were not subsequently included in AMYPAD, resulting in a replication sample of 927 subjects. General characteristics of the selected EPAD participants for replication are given in Supplementary Table 2.

**Supplementary Table 2.** Demographics and clinical characteristics of EPAD participants used for replication.

|  | Overall  (N=927) | CDR = 0  (N=646) | CDR = 0.5  (N=274) |
| --- | --- | --- | --- |
| Age, years. mean (SD) | 65.21 (7.62) | 63.91 (7.17) | 68.13 (7.81) |
| Sex, male. N (%) | 392 (42.3) | 254 (39.3) | 137 ( 50.0) |
| MMSE, score. mean (SD) | 28.29 (2.06) | 28.88 (1.27) | 26.92 (2.76) |
| Education, years. mean (SD) | 14.37 (3.68) | 14.93 (3.46) | 13.03 (3.85) |
| CSF, Aβ1-42. mean (SD) | 1386.80 (764.55) | 1475.43 (740.20) | 1174.25 (785.82) |
| CSF, P-Tau181. mean (SD) | 19.66 (10.30) | 17.46 (7.67) | 25.06 (13.46) |
| CSF, T-Tau. mean (SD) | 224.94 (99.11) | 204.51 (78.10) | 275.02 (124.31) |
| APOE ε4, carrier. N (%) | 349 (39.3) | 228 (36.5) | 120 ( 46.2) |

# Supplementary Results

## Subtype definition and sensitivity

As described in the main text, we found that two subtypes yielded the best fit of the NMF model based on the chosen criteria. Fit indices, as described in the Supplementary Methods, are reported in Supplementary Table 3.

**Supplementary Table 3.** Non-negative matrix factorization fit indices in the discovery cohort.

| Clusters | Coph. Coeff | Sil. Coef | RSS (norm) | RSS (rand) | RSS change (norm) | RSS change (rand) |
| --- | --- | --- | --- | --- | --- | --- |
| 2 | 0.936801 | 0.99 | 1812.462 | 3463.111 | 451.623 | 55.808 |
| 3 | 0.900928 | 0.624171 | 1683.734 | 3404.962 | 128.7279 | 58.14877 |
| 4 | 0.851089 | 0.415451 | 1590.128 | 3342.961 | 93.60663 | 62.00093 |
| 5 | 0.837029 | 0.447046 | 1489.014 | 3280.314 | 101.1142 | 62.64749 |
| 6 | 0.809378 | 0.365315 | 1404.155 | 3233.961 | 84.85834 | 46.3532 |

As shown in the table, also the three subtypes solution had a good fit to the data. However, we based our decision to proceed with 2 on several reasons. Firstly, in cases where more solutions show a good fit to the data, one tends to prefer simpler models: the model with only 2 subtypes in this case. The 2 subtype model also showed better fit overall, with highest cophonetic and silhouette coefficient, and larger changes in RSS, also compared to the random solution. Moreover, recent literature on cohorts with similar characteristics has shown that 2 subtypes can be consistently identified in the data.[(Baumeister et al. 2024)](https://paperpile.com/c/jYy4u9/Hn516) In addition, when fitting the validation algorithm (SuStaIn) we also found 2 subtypes to be the best fitting number. Finally, we performed a sensitivity analysis using three subtypes. In this analysis, the AD-typical pattern was splitted in 2 subtypes, one more medial and one more lateral temporal (Supplementary Figure 1A). While this could also be in line with previous literature, the stability analysis showed that these two subtypes were less stable over time and tended to switch from one to the other (Supplementary Figure 1 B-C). This suggests that this subdivision may reflect stage-related subtypes, with the limbic-predominant form representing a later manifestation of the typical AD subtype, sharing a common underlying pathophysiology.


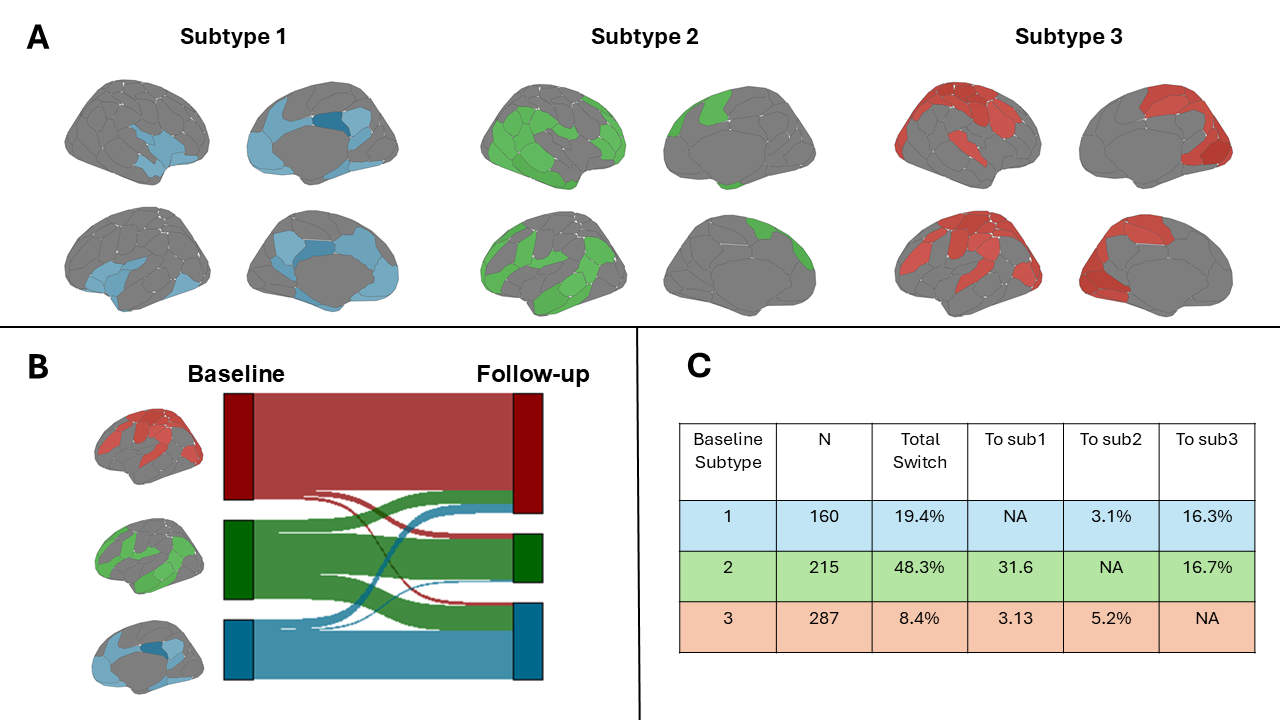


**Supplementary Figure 1.** Results of NMF model when using three clusters (A), and stability of identified subtypes over time (BC).

## Subtype Characterization

**Supplementary Table 4.** Results of logistic regression model predicting Subtype assignment (1 vs 2). Predictors included centiloid, APOE e4 carriership, age and sex. Models were corrected for subtype probability.

|  | Estimate | Std. Error | z value | Pr(>\|z\|) |
| --- | --- | --- | --- | --- |
| Centiloid | -0.00422 | 0.001996 | -2.114 | 0.034480 |
| APOE e4 | -0.4179 | 0.123407 | -3.386 | 0.000708 |
| Age | -0.018255 | 0.007231 | -2.525 | 0.011586 |
| Sex (male) | 0.149162 | 0.114056 | 1.308 | 0.190943 |

**Supplementary Table 5.** Results of linear mixed effects models. Coefficients of the interaction between subtype and time in predicting centiloid and 4 cognitive outcomes as described in the methods. Estimated marginal trends are reported for each subtype and each outcome.

| Outcome | Subtype X time (years) | | Subtype1 X time (years) | | Subtype2 X time (years) | |
| --- | --- | --- | --- | --- | --- | --- |
|  | β | p-value | β | p-value | β | p-value |
| Centiloid | 0.472 | 0.0006 | 1.80 | <0.0001 | 1.33 | <0.0001 |
| Memory DR | -0.0301 | 0.1110 | -0.0184 | 0.2210 | 0.0118 | 0.3087 |
| Memory IR | -0.0191 | 0.2999 | -0.01621 | 0.2651 | 0.00291 | 0.7984 |
| DS Forward | 0.0816 | 0.0408 | -0.0615 | 0.0410 | -0.1431 | <0.0001 |
| DS Backward | 0.138 | 0.0002 | 0.0933 | 0.0008 | -0.0449 | 0.0768 |

**Supplementary Table 6.** Results of linear mixed effects models investigating individuals NMF scores within subtypes associated with longitudinal outcomes. For each subtype, an interaction between NMF scores and time was used in the models predicting the 4 cognitive outcomes. NMF scores were used continuously. Median-split was used for visualization purposes in the main text.

| Outcome | Subtype 1  NMF score X time | | Subtype 2  NMF score X time | |
| --- | --- | --- | --- | --- |
|  | β | p-value | β | p-value |
| Memory DR | -8.2985 | 0.008 | 8.7794 | 0.531 |
| Memory IR | -0.4124 | 0.0829 | -0.4982 | 0.172 |
| DS Forward | -0.4341 | 0.4131 | -0.4873 | 0.56995 |
| DS Backward | 0.0653 | 0.90117 | -1.223 | 0.13817 |

## Disproportional effects of cognitive tests on longitudinal changes

In the main text, we described how the two subtypes seem to have distinct longitudinal trajectories in their cognitive performance. Specifically, subtype 1 showed a faster decrease in memory-related score (immediate and delayed recall) while subtype 2 had a larger impact on digit span scores (see Figure 3). As discussed in the main text and in the “Neuropsychological Evaluation” section of this document, the memory scores were composed by a mixture of different tests that were performed in different cohorts. While we tried to harmonize the scores by using a cohort-specific scaling procedure, it remains important to evaluate whether certain tests have disproportional effects and drive the association observed in the main analysis.

To address this issue, we repeated the linear mixed effects models by iteratively removing one test from the investigated sample (i.e. removing one cohort). Following the procedures used for the main analysis, linear mixed effects models included the cognitive scores as outcomes. Predictors included participants' subtype assignment and its interaction with time. Models also included the correction for age, sex, global baseline CL, baseline NMF probability and a random intercept on the participants. Within subtypes, we also studied the impact of having higher NMF scores on longitudinal outcomes (NMF score-by-time interaction).

Supplementary Table 7 reports the model coefficients. As in Figure 3 of the main paper, we report the p-value of the interaction of subtype by time, and the beta coefficients for each subtype. Moreover, within each subtypes we also report the p-value of the interaction of NMF probability with time.

The results demonstrate replicable effects across different iterations. In Delayed recall memory scores, subtype one shows negative slopes and significant interaction between NMF scores and time in most tests, to the exclusion of FCRST.

**Supplementary Table 7.** Results of linear models iteratively excluding memory tests.

|  | **Subtype-by-Time Interaction** | | | **Subtype 1: NMF Scores-by-Time Interaction** | **Subtype 2: NMF Scores-by-Time Interaction** |
| --- | --- | --- | --- | --- | --- |
| **Delayed Recall** | **Sub1 Beta** | **Sub 2 Beta** | **P-value interaction** | **P-value** | **P-value** |
| Leave out: CERAD | -0.02 | 0.01 | 0.096 | 0.006 | 0.491 |
| Leave out: FCRST | 0.02 | 0.01 | 0.678 | 0.427 | 0.506 |
| Leave out: RAVLT | -0.05 | 0.01 | 0.057 | 0.201 | 0.615 |
| Leave out: RBANS | -0.03 | 0.01 | 0.047 | 0.016 | 0.485 |
| Leave out: WMS III | -0.02 | 0.01 | 0.221 | 0.007 | 0.238 |
| **Immediate Recall** |  |  |  |  |  |
| Leave out: CERAD | -0.02 | 0.01 | 0.252 | 0.089 | 0.172 |
| Leave out: FCRST | 0.03 | 0.01 | 0.392 | 0.800 | 0.163 |
| Leave out: RAVLT | -0.03 | -0.02 | 0.391 | 0.473 | 0.332 |
| Leave out: RBANS | -0.02 | 0.01 | 0.195 | 0.092 | 0.421 |
| Leave out: WMS III | -0.02 | 0.01 | 0.125 | 0.123 | 0.158 |

##

## Determinants of Subtype stability over time

|  | OR | 2.5% | 97.5% | P-value |
| --- | --- | --- | --- | --- |
| Sex | 1.0991 | 0.6333 | 1.9263 | 0.738 |
| Time | 0.9755 | 0.8489 | 1.1272 | 0.731 |
| NMF probability | 1.0278 | 1.0205 | 1.0362 | <0.0001 |
| Age | 1.0092 | 0.9722 | 1.0496 | 0.637 |

**Supplementary Table 8.** Results of logistic regression models predicting subtype stability (stable vs unstable).

##

## Effect of time on thickness within subtypes

Supplementary Table 9 reports the p-values of the linear mixed effects models investigating the effect of subtype assignment on longitudinal regional thickness values (Subtype x Time interaction). Moreover, it reports the values of the effect of time within each subtype, computed with estimated marginal means (emmeans).

## Thickness Subtypes and Longitudinal Progression Using Deskan Killiany Atlas

Subtypes' replicability across a series of conditions, including distinct brain parcellations, is a crucial condition for the generalizability of our results. Moreover, the Schaefer atlas is a parcellation based on the functional profile of regions, thus possibly biasing subsequent analysis on network-determined progression of thinning. For this reason, we performed a sensitivity analysis using a different parcellation, namely the Desikan Killiany (DK) atlas [(Desikan et al. 2006)](https://paperpile.com/c/jYy4u9/quT9). Cortical gray matter thickness volumes were extracted for the 68 regions of interest of the DK atlas. NMF, linear mixed models for longitudinal thinning, and coordinated deformation models for this new set of data were performed using the exact same procedures as in the main analysis (with the Schaefer atlas).

NMF cluster optimization parameters are shown in Supplementary Table 10. Similarly to our main analysis, the best number of fitting clusters was 2.

**Supplementary Table 10.** Non-negative matrix factorization fit indices in the discovery cohort using the Desikan Killiany atlas.

| Clusters | Coph. Coeff | Sil. Coef | RSS change (norm) | RSS change (rand) |
| --- | --- | --- | --- | --- |
| 2 | 0.96022889 | 1 | 569.474939 | 209.718581 |
| 3 | 0.92140178 | 0.81812268 | 175.82523 | 180.49686 |
| 4 | 0.86011785 | 0.62023157 | 132.41552 | 171.112449 |
| 5 | 0.87310424 | 0.48441857 | 77.6957428 | 156.054629 |
| 6 | 0.86732287 | 0.3897627 | 83.0308151 | 105.307823 |

Supplementary Figure 2A shows the regional assignments and loadings when fitting the NMF model with two substypes. In line with our main results, the first subtype was characterized by the involvement of limbic and temporal areas. The second subtype had diffuse regional vulnerability sparing the medial-temporal lobe. Of the 601 participants assigned to subtype 1 when using the Schaefer atlas (main results), 513 (85.4%) were also assigned to subtype 1 when using the DK atlas. Of the 722 participants assigned to subtype 2 when using the Schaefer atlas (main results), 655 (90.7%) were also assigned to subtype 2 when using the DK atlas.

The results of the linear mixed effect models are illustrated in Supplementary Figure 2B. Similarly to our main analysis, subtype 1 showed faster reductions of thickness over time in lateral superior temporal regions and posterior medial regions, while subtype 2 had faster reductions of thickness over time mostly in dorsal regions, including dorsal parietal and frontal regions, and also in lateral temporal regions.

Supplementary Figure 2C shows the results of the coordinated deformation models performed on this new set of data. Generally, the results from our main analysis were confirmed, as such all types of connectivity were significantly predicting thinning progression in subtype 1 and morphological similarity was significantly predicting thinning progression in subtype 2. Interestingly, for subtype 1 however SC had a stronger correlation compared to others, and was also significant in the subtype 2 models, even though with smaller effects compared to morphological similarity.

*
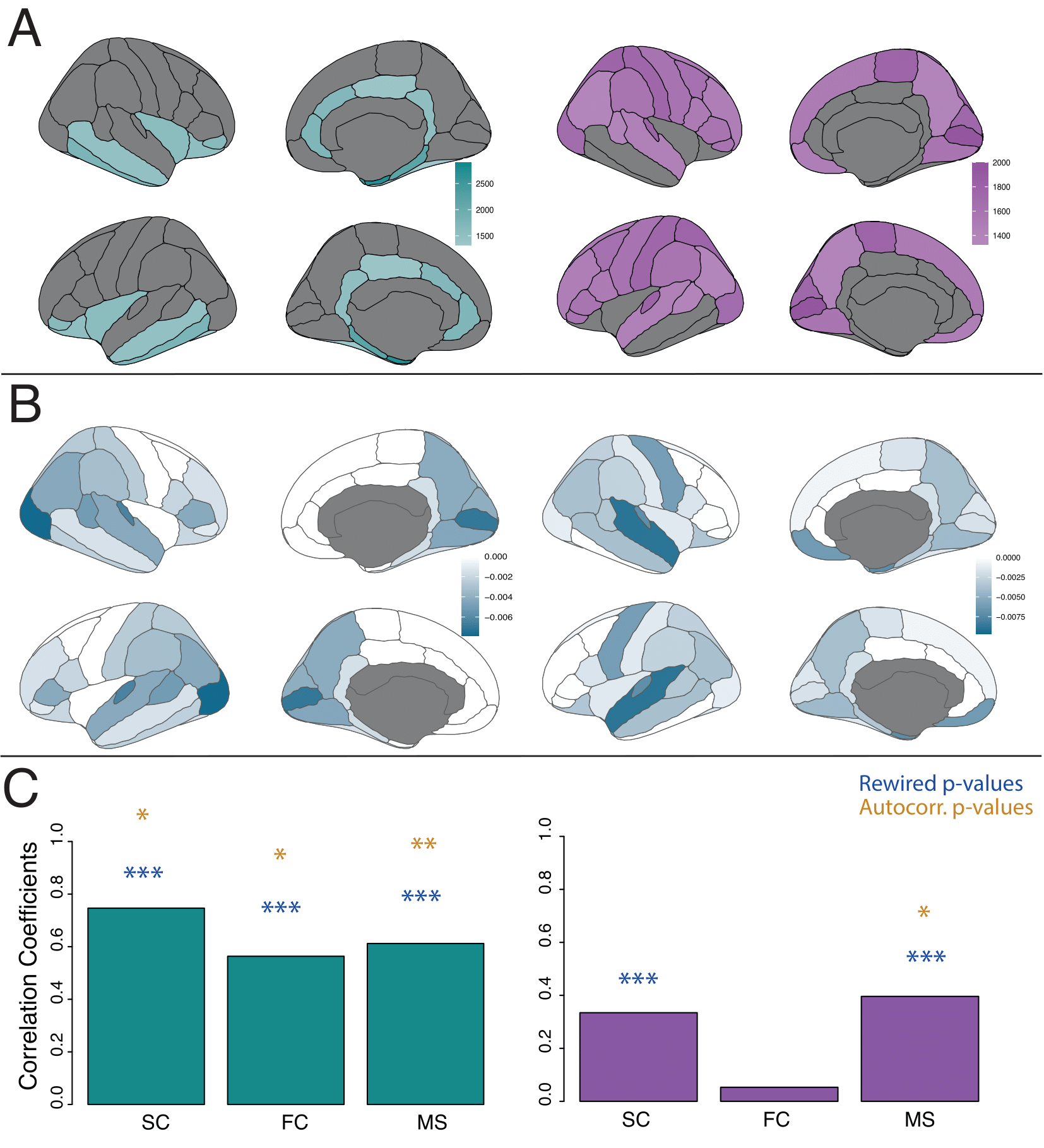
*

**Supplementary Figure 2.** A) Regional thickness subtypes obtained with NMF when using the DK atlas parcellation. B) Effect of time on regional thickness within each subtype. C). Results of the coordinated deformation models.

## Thickness Subtypes on site-harmonized thickness values

We further aimed at evaluating whether scanner site differences had an impact on our results. To do so, we performed a statistical harmonization of the gray matter thickness data and re-run the NMF analysis. Statistical harmonization of regional gray matter thickness values was performed through neuroComabat.[(Fortin et al. 2018)](https://paperpile.com/c/jYy4u9/tz2Nn) By taking advantage of ComBat features, we harmonized regional thickness values between scanning-sites while retaining the variability due to biologically-relevant variables, including age, sex and amyloid status. Supplementary Figure 3A shows the distribution of thickness values from a specific region (left Precuneus) across sites before and after ComBat harmonization. To confirm our results, we re-run the full NMF pipeline on the harmonized thickness data, including cluster number optimization. As shown in Supplementary Table 11, the solution with 2 clusters was the best fitting model, confirming our main results. When fitting the NMF model using a 2 cluster-solution on the harmonized thickness data we obtained two subtypes that strongly resembled the ones reported in the main text. Supplementary Figure 3B shows the cortical maps of the 2 identified subtypes. The first subtype was characterized by the involvement of limbic and temporal areas. The second subtype had diffuse regional vulnerability sparing the medial-temporal lobe. We then evaluated the overlap between these subtypes and the one previously identified, both in terms of regional and participant assignment. Supplementary Figure 3C shows the overlap in participant assignment between the original NMF and the harmonized thickness NMF. Of the 601 participants originally assigned to subtype 1, 551 (91.7%) were assigned to the same subtype in the harmonized NMF. Of the 722 participants originally assigned to subtype 2, 547 (75.8%) were assigned to the same subtype in the harmonized NMF. These results suggest high stability in the assignment of participants to either subtype, independently of the harmonization step. Supplementary Figure 3D shows the correlations between NMF loadings (contribution of each region to each subtype) in the original and harmonized NMF. For both subtypes, we found high correlation in regional loadings (>0.9), demonstrating high agreement in regional assignment.

**Supplementary Table 11.** Non-negative matrix factorization fit indices in the discovery cohort after neuroCombat harmonization.

| Clusters | Coph. Coeff | Sil. Coef | RSS change (norm) | RSS change (rand) |
| --- | --- | --- | --- | --- |
| 2 | 0.9351 | 1.000 | 300.263 | 46.170 |
| 3 | 0.9076 | 0.700 | 102.697 | 55.002 |
| 4 | 0.8605 | 0.319 | 66.884 | 34.566 |
| 5 | 0.8349 | 0.361 | 81.683 | 44.514 |
| 6 | 0.8218 | 0.281 | 73.683 | 43.093 |

*
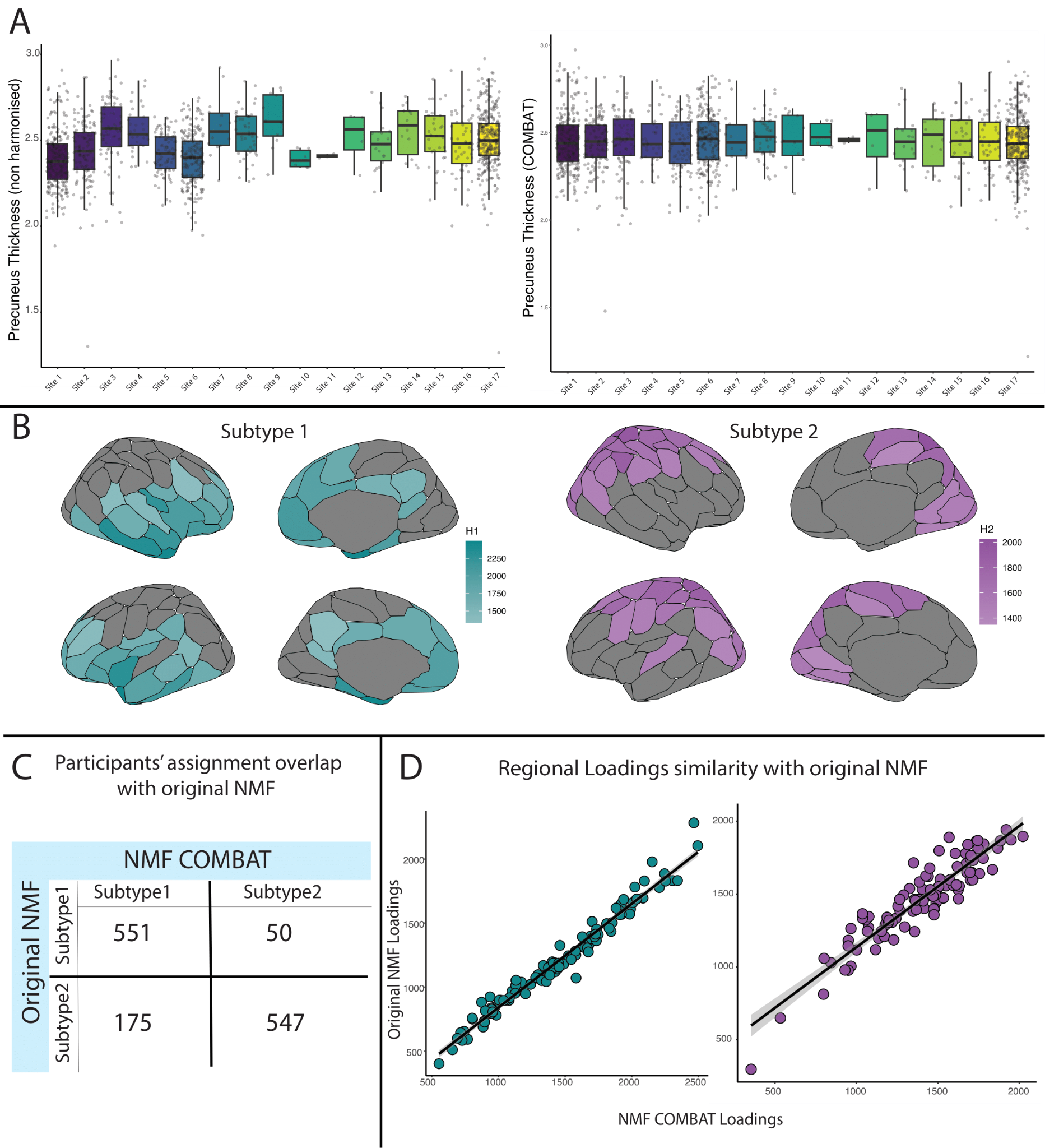
*

**Supplementary Figure 3.** Results of NMF after neuroCombat harmonization. A) boxplot showing average thickness values in the precuneus before and after harmonization. B) Regional thickness subtypes found with NMF after neuroCombat harmonization. C) Agreement between participants’ subtype assignment in the original vs the harmonized NMF. D) Agreement in regional loadings in the original vs the harmonized NMF.

## Thickness Subtypes in age- and sex- corrected thickness values

To ensure that age was not the main driver of the observed results and that the two subtypes were not the expression of two different stages of the brain aging process, we performed a sensitivity analysis on age- and sex-corrected regional gray matter thickness data. Specifically, we performed linear models predicting regional cortical thickness values (dependent variables) with participants’ age and sex (independent variables) and computed models’ residuals. We then performed the NMF clustering algorithm on the obtained regional residuals, following the same procedures used for the main analysis. Supplementary Figure 4A shows the regions assigned to the two identified subtypes. Similarly to our main analysis, the first subtype shows limbic and temporal involvement while the second subtype demonstrates diffuse regional involvement sparing the medio-temporal lobe. Supplementary Figure 4B shows the agreement in participants’ subtype assignment between our original (uncorrected) and the age- and sex-corrected NMF results. Of the 601 participants originally assigned to subtype 1, 566 (94.2%) were also assigned to subtype 1 when correcting for age and sex. Of the 722 participants originally assigned to subtype 2, 675 (93.5%) were assigned to the same subtype when correcting for age and sex. Finally, Supplementary Figure 4C we report the correlation between regional loadings in the original and in the age-corrected NMF run. For both subtypes, regional loadings had very high correlations (r >0.9) between the two NMF runs.

*
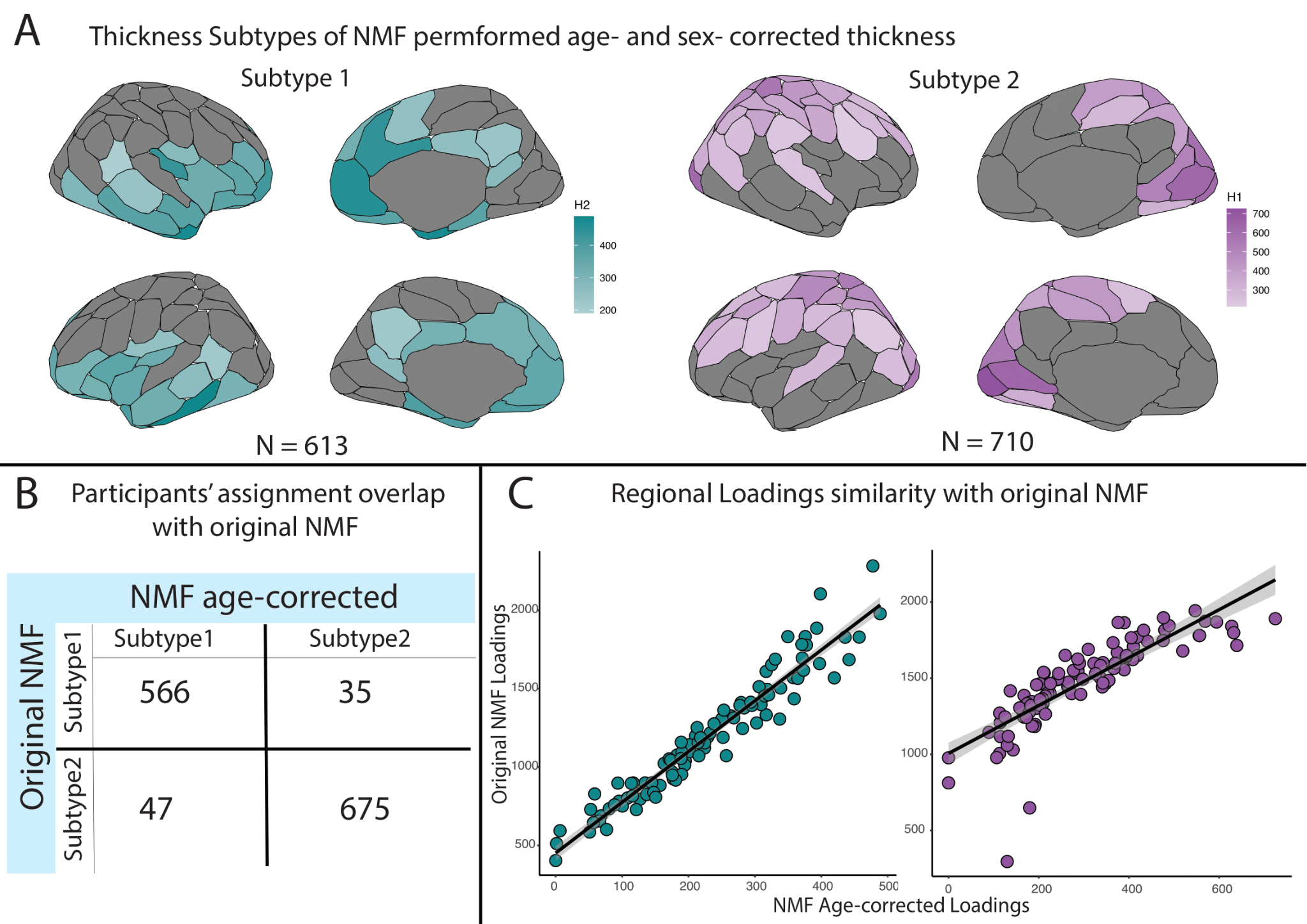
*

**Supplementary Figure 4.** Results of NMF after regressing out age and sex from thickness values. A) Regional thickness subtypes found with NMF age- and sex- corrected values. B) Agreement between participants’ subtype assignment in the original vs the corrected NMF. C) Agreement in regional loadings in the original vs the corrected NMF.

## Thickness Subtypes in Amyloid positive Participants

To evaluate whether the two identified subtypes were mostly driven by the presence of amyloid, more abundant in the limbic subtype, and were therefore the expression of the presence of AD pathology, we replicated our analysis by only including amyloid positive individuals (N=299). NMF cluster optimization parameters are shown in Supplementary Table 12. Similarly to our main analysis, the best number of fitting clusters was 2.

**Supplementary Table 12.** Non-negative matrix factorization on only A+ participants fit indices.

| Clusters | Coph. Coeff | Sil. Coef | RSS change (norm) | RSS change (rand) |
| --- | --- | --- | --- | --- |
| 2 | 0.9111 | 1 | 97.105 | 19.570 |
| 3 | 0.8945 | 0.7464 | 32.408 | 23.139 |
| 4 | 0.812 | 0.5668 | 27.134 | 22.132 |
| 5 | 0.7989 | 0.2730 | 24.953 | 18.579 |
| 6 | 0.8169 | 0.2329 | 21.668 | 21.101 |

Supplementary Figure 5A shows the regional assignments and loadings when fitting the NMF model with two subtypes. In line with our main results, the first subtype was characterized by the involvement of limbic and temporal areas. The second subtype had diffuse regional vulnerability sparing the medial-temporal lobe.

Supplementary Figure 5B shows the agreement in participants’ assignment to subtypes between the original NMF (on all participants) and the NMF on exclusively amyloid positive participants. Of the 159 participants that were originally assigned to subtype 1, 136 (85.5 %) were also assigned to this subtype in this analysis. All 140 (100%) participants originally assigned to subtype 2 were also assigned to the same subtype in this analysis.

When evaluating regional loadings to each subtype, the original NMF run and the NMF on exclusively A+ participants showed very high correlations (>0.90) in both subtypes (Supplementary Figure 5C).


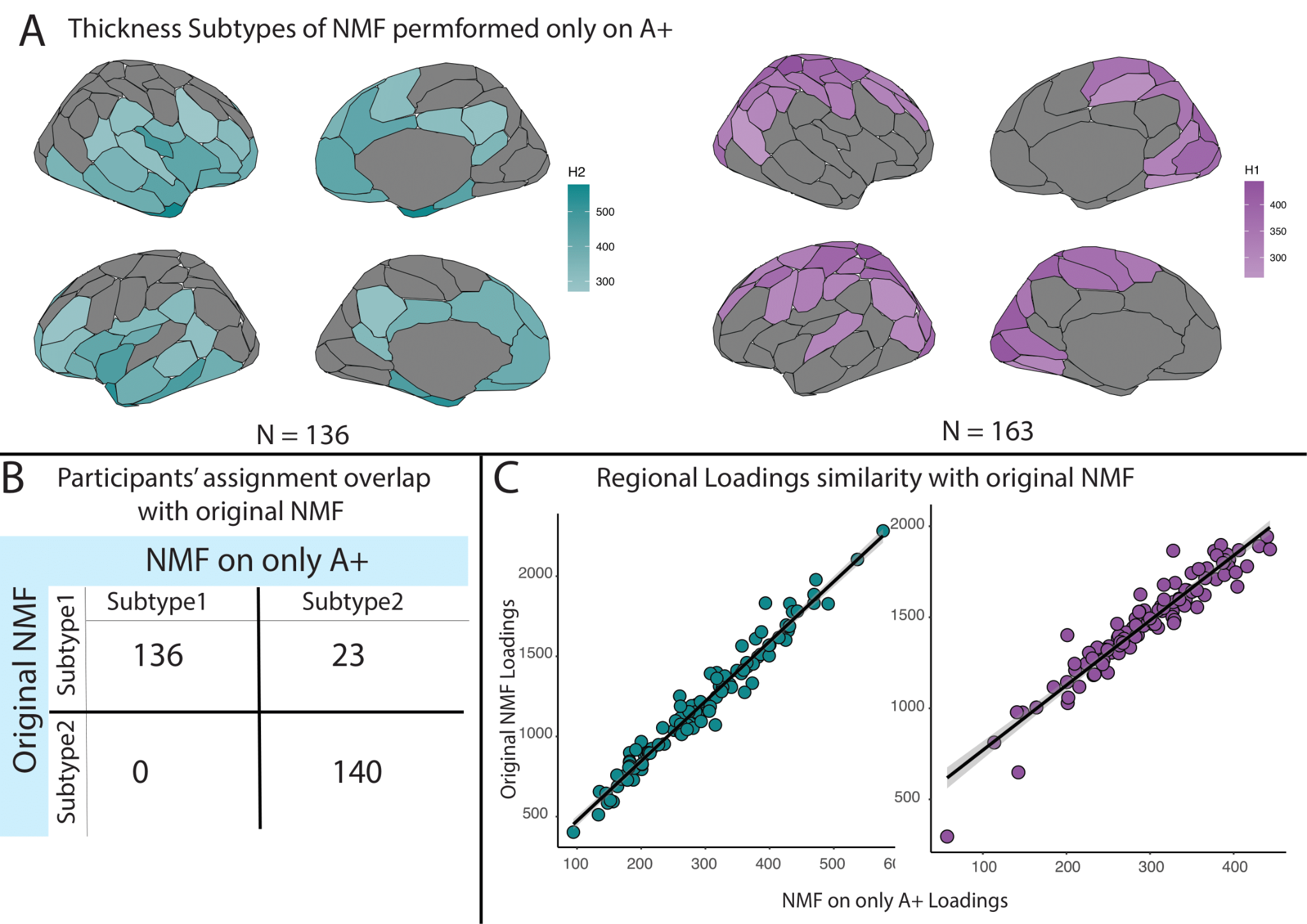


**Supplementary Figure 5.** Results of NMF when performed exclusively on A+ participants. A) Regional thickness subtypes found A+ participants. B) Agreement between participants’ subtype assignment in the original vs the A+ NMF. C) Agreement in regional loadings in the original vs the A+ NMF.

##

## SuStaIn Results

##
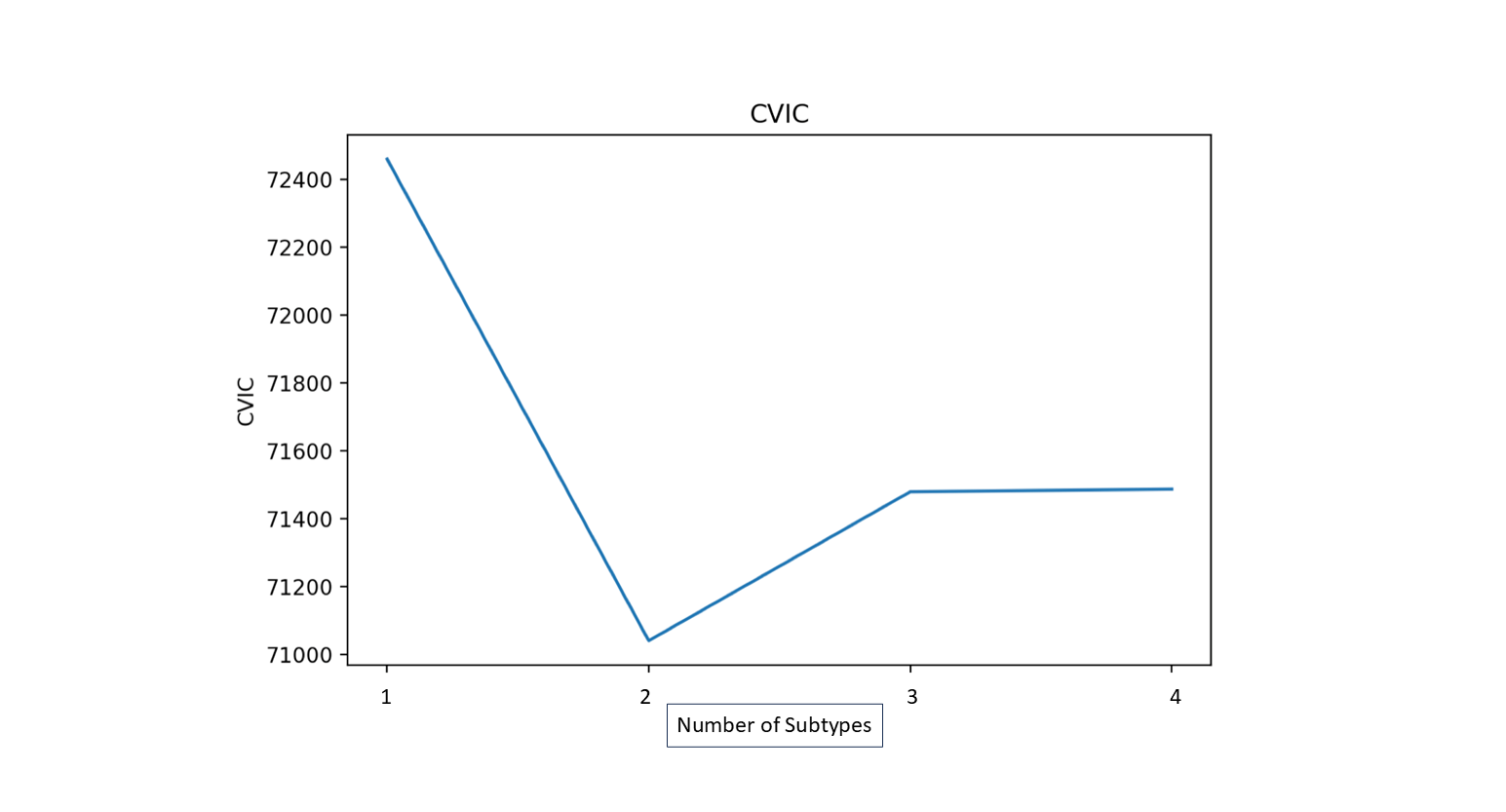


**Supplementary Figure 6.** Cross-validation information criteria (CVIC) for each number of subtypes in the SuStain model

## NMF in the replication Cohort

**Supplementary Table 13.** Non-negative matrix factorization fit indices in the replication cohort.

| Clusters | Coph. Coeff | Sil. Coef | RSS (norm) | RSS (rand) | RSS change (norm) | RSS change (rand) |
| --- | --- | --- | --- | --- | --- | --- |
| 2 | 0.928156 | 1 | 1388.894 | 2372.458 | 184.956 | 44.435 |
| 3 | 0.877251 | 0.680574 | 1272.739 | 2336.793 | 116.1545 | 35.66519 |
| 4 | 0.83267 | 0.350598 | 1197.098 | 2305.028 | 75.64096 | 31.76432 |
| 5 | 0.849916 | 0.392083 | 1130.094 | 2259.826 | 67.0047 | 45.20215 |
| 6 | 0.823052 | 0.30524 | 1074.439 | 2222.232 | 55.65506 | 37.59384 |

# Sustain with different Z threshold

To evaluate the impact of the selected threshold on the observed output of SuStaIn, we run a sensitivity analysis increasing the threshold to z=1.96, to reflect pathological changes. The results of this analysis are shown in Supplementary Figure 6. The upper row shows the CVIC and the log-likelihood of Sustain models (z threshold=1.96) with an increasing number of subtypes. As shown, both indices show a great improvement from the 1- to the 2-subtypes solution, and then stabilize with minimal improvements. In this case, the model with lower complexity and greater biological plausibility is preferred [(Pauler, Wakefield, and Kass 1999)](https://paperpile.com/c/jYy4u9/LjAm), thus confirming our main results.

When looking at the patterns of cortical thinning of the two subtypes (lower row), these were substantially unchanged from the previous analysis with threshold z=1.

The first one had initial thinning in occipital areas, followed by dorso-parietal, and eventually frontal areas the second SuStaIn subtype cortical thinning progressed from medial temporal, to medial frontal and lateral temporal regions, with a pattern similar to the NMF subtype 1.

When looking at the agreement in participants assignment, of the 757 participants assigned to the first subtype in the z=1 analysis , 732 (96.7%) were assigned to the same subtype in the z=1.96 analysis. Of the 566 participants assigned to the second subtype in the z=1 analysis, 560 (98.9%) were assigned to the same subtype in the z=1.96 analysis.


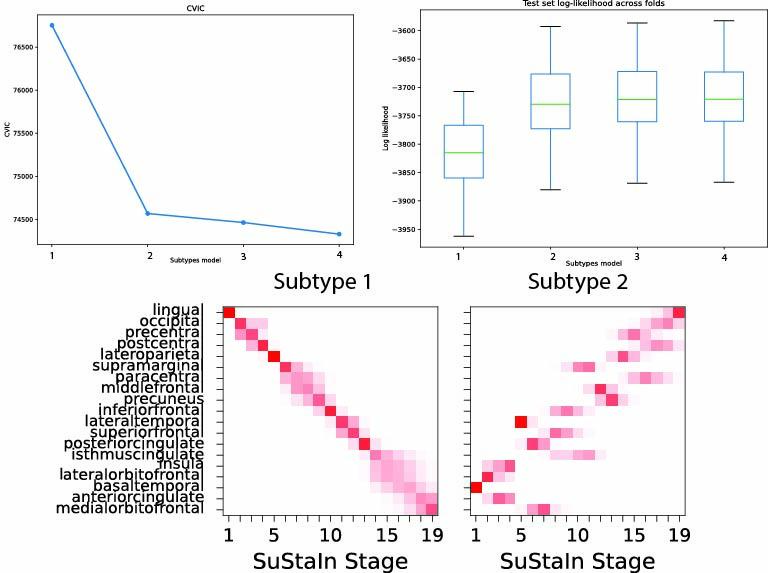


**Supplementary Figure 7.** Results of SuStaIn analysis when using z threshold of 1.96. Upper row: CVIC and log-likelihood of models with increasing number of clusters. Lower row: Regional progression of cortical thinning within the two subtypes.

#

# References

[Alegret, Montserrat, Ana Espinosa, Sergi Valero, Georgina Vinyes-Junqué, Agustín Ruiz, Isabel Hernández, Maitee Rosende-Roca, et al. 2013. “Cut-off Scores of a Brief Neuropsychological Battery (NBACE) for Spanish Individual Adults Older than 44 Years Old.” *PloS One* 8 (10): e76436.](http://paperpile.com/b/jYy4u9/t4hdV)

[Balusu, Sriram, Roman Praschberger, Elsa Lauwers, Bart De Strooper, and Patrik Verstreken. 2023. “Neurodegeneration Cell per Cell.” *Neuron* 111 (6): 767–86.](http://paperpile.com/b/jYy4u9/EaxiZ)

[Baumeister, Hannah, Jacob W. Vogel, Philip S. Insel, Luca Kleineidam, Steffen Wolfsgruber, Melina Stark, Helena M. Gellersen, et al. 2024. “A Generalizable Data-Driven Model of Atrophy Heterogeneity and Progression in Memory Clinic Settings.” *Brain: A Journal of Neurology*, April. https://doi.org/](http://paperpile.com/b/jYy4u9/Hn516)[10.1093/brain/awae118](http://dx.doi.org/10.1093/brain/awae118)[.](http://paperpile.com/b/jYy4u9/Hn516)

[Bellenguez, Céline, Fahri Küçükali, Iris E. Jansen, Luca Kleineidam, Sonia Moreno-Grau, Najaf Amin, Adam C. Naj, et al. 2022. “New Insights into the Genetic Etiology of Alzheimer’s Disease and Related Dementias.” *Nature Genetics* 54 (4): 412–36.](http://paperpile.com/b/jYy4u9/l0Fl1)

[Collij, Lyduine E., Gemma Salvadó, Viktor Wottschel, Sophie E. Mastenbroek, Pierre Schoenmakers, Fiona Heeman, Leon Aksman, et al. 2022. “Spatial-Temporal Patterns of β-Amyloid Accumulation: A Subtype and Stage Inference Model Analysis.” *Neurology* 98 (17): e1692–1703.](http://paperpile.com/b/jYy4u9/wSPM6)

[Desikan, Rahul S., Florent Ségonne, Bruce Fischl, Brian T. Quinn, Bradford C. Dickerson, Deborah Blacker, Randy L. Buckner, et al. 2006. “An Automated Labeling System for Subdividing the Human Cerebral Cortex on MRI Scans into Gyral Based Regions of Interest.” *NeuroImage* 31 (3): 968–80.](http://paperpile.com/b/jYy4u9/quT9)

[Esteban, Oscar, Christopher J. Markiewicz, Ross W. Blair, Craig A. Moodie, A. Ilkay Isik, Asier Erramuzpe, James D. Kent, et al. 2019. “fMRIPrep: A Robust Preprocessing Pipeline for Functional MRI.” *Nature Methods* 16 (1): 111–16.](http://paperpile.com/b/jYy4u9/mvBFE)

[Fortin, Jean-Philippe, Nicholas Cullen, Yvette I. Sheline, Warren D. Taylor, Irem Aselcioglu, Philip A. Cook, Phil Adams, et al. 2018. “Harmonization of Cortical Thickness Measurements across Scanners and Sites.” *NeuroImage* 167 (February):104–20.](http://paperpile.com/b/jYy4u9/tz2Nn)

[Gaujoux, Renaud, and Cathal Seoighe. 2010. “A Flexible R Package for Nonnegative Matrix Factorization.” *BMC Bioinformatics* 11 (1): 367.](http://paperpile.com/b/jYy4u9/KwhjA)

[Grober, Ellen, and Herman Buschke. 1987. “Genuine Memory Deficits in Dementia.” *Developmental Neuropsychology* 3 (1): 13–36.](http://paperpile.com/b/jYy4u9/2WTgF)

[Lezak, M. D. 1998. “Tests: One Old, One New (mostly). Rey Auditory and Verbal Learning Test. A Handbook, by Michael Schmidt. 1996. Los Angeles, CA: Western Psychological Services. 137 Pp., 49.50. The Camden Memory Tests, by Elizabeth K. Warrington. 1996. Hove, U.k.: Psychology Press (erlbaum, Taylor & Francis). Manual. 16 Pp., 9.95. ISBN: 0-86377-379-6. Short Recognition Memory Test for Words, 30.00. ISBN: 0-86377-429-6; Short Recognition Memory Test for Faces, 35.00. ISBN: 0-86377-430-X; Paired Associate Learning Test, 35.00. ISBN: 0-86377-428-8; Topographical Recognition Memory Test, 85.00. ISBN: 0-86377-427-X; Pictorial Recognition Test, $85.00. ISBN: 0-86377-426-1.” *Journal of the International Neuropsychological Society: JINS* 4 (4): 410–14.](http://paperpile.com/b/jYy4u9/bTBOD)

[Mishra, Aniket, Rainer Malik, Tsuyoshi Hachiya, Tuuli Jürgenson, Shinichi Namba, Daniel C. Posner, Frederick K. Kamanu, et al. 2022. “Stroke Genetics Informs Drug Discovery and Risk Prediction across Ancestries.” *Nature* 611 (7934): 115–23.](http://paperpile.com/b/jYy4u9/nnCZh)

[Morris, J. C., A. Heyman, R. C. Mohs, J. P. Hughes, G. van Belle, G. Fillenbaum, E. D. Mellits, and C. Clark. 1989. “The Consortium to Establish a Registry for Alzheimer’s Disease (CERAD). Part I. Clinical and Neuropsychological Assessment of Alzheimer's Disease.” *Neurology* 39 (9): 1159–65.](http://paperpile.com/b/jYy4u9/HsvCn)

[Murman, Daniel L. 2015. “The Impact of Age on Cognition.” *Seminars in Hearing* 36 (3): 111–21.](http://paperpile.com/b/jYy4u9/oLpCL)

[Nelson, Peter T., David W. Fardo, Xian Wu, Khine Zin Aung, Matthew D. Cykowski, and Yuriko Katsumata. 2024. “Limbic-Predominant Age-Related TDP-43 Encephalopathy (LATE-NC): Co-Pathologies and Genetic Risk Factors Provide Clues about Pathogenesis.” *Journal of Neuropathology and Experimental Neurology* 83 (6): 396–415.](http://paperpile.com/b/jYy4u9/IuGvV)

[Pascual-Montano, Alberto, J. M. Carazo, Kieko Kochi, Dietrich Lehmann, and Roberto D. Pascual-Marqui. 2006. “Nonsmooth Nonnegative Matrix Factorization (nsNMF).” *IEEE Transactions on Pattern Analysis and Machine Intelligence* 28 (3): 403–15.](http://paperpile.com/b/jYy4u9/MFTPm)

[Pauler, Donna K., Jonathan C. Wakefield, and Robert E. Kass. 1999. “Bayes Factors and Approximations for Variance Component Models.” *Journal of the American Statistical Association* 94 (448): 1242–53.](http://paperpile.com/b/jYy4u9/LjAm)

[Persyn, Elodie, Ken B. Hanscombe, Joanna M. M. Howson, Cathryn M. Lewis, Matthew Traylor, and Hugh S. Markus. 2020. “Genome-Wide Association Study of MRI Markers of Cerebral Small Vessel Disease in 42,310 Participants.” *Nature Communications* 11 (1): 2175.](http://paperpile.com/b/jYy4u9/fouWe)

[Ritchie, C. W., G. Muniz-Terrera, M. Kivipelto, A. Solomon, B. Tom, and J. L. Molinuevo. 2020. “The European Prevention of Alzheimer’s Dementia (EPAD) Longitudinal Cohort Study: Baseline Data Release V500.0.” *The Journal of Prevention of Alzheimer’s Disease* 7 (1): 8–13.](http://paperpile.com/b/jYy4u9/CJC2n)

[Schott, Jonathan M., Sebastian J. Crutch, Minerva M. Carrasquillo, James Uphill, Tim J. Shakespeare, Natalie S. Ryan, Keir X. Yong, et al. 2016. “Genetic Risk Factors for the Posterior Cortical Atrophy Variant of Alzheimer’s Disease.” *Alzheimer’s & Dementia: The Journal of the Alzheimer's Association* 12 (8): 862–71.](http://paperpile.com/b/jYy4u9/lo95z)

[Sebenius, Isaac, Jakob Seidlitz, Varun Warrier, Richard A. I. Bethlehem, Aaron Alexander-Bloch, Travis T. Mallard, Rafael Romero Garcia, Edward T. Bullmore, and Sarah E. Morgan. 2022. “MIND Networks: Robust Estimation of Structural Similarity from Brain MRI.” https://doi.org/](http://paperpile.com/b/jYy4u9/WErsz)[10.1101/2022.10.12.511922](http://dx.doi.org/10.1101/2022.10.12.511922)[.](http://paperpile.com/b/jYy4u9/WErsz)

[Smith, Robert E., Jacques-Donald Tournier, Fernando Calamante, and Alan Connelly. 2015. “SIFT2: Enabling Dense Quantitative Assessment of Brain White Matter Connectivity Using Streamlines Tractography.” *NeuroImage* 119 (October):338–51.](http://paperpile.com/b/jYy4u9/LnoNA)

[Vogel, Jacob W., Alexandra L. Young, Neil P. Oxtoby, Ruben Smith, Rik Ossenkoppele, Olof T. Strandberg, Renaud La Joie, et al. 2021. “Four Distinct Trajectories of Tau Deposition Identified in Alzheimer’s Disease.” *Nature Medicine* 27 (5): 871–81.](http://paperpile.com/b/jYy4u9/hPMMA)

[Yang, Chengwu, Elizabeth Garrett-Mayer, Jay S. Schneider, Stephen M. Gollomp, and Barbara C. Tilley. 2009. “Repeatable Battery for Assessment of Neuropsychological Status in Early Parkinson’s Disease: RBANS in Early PD.” *Movement Disorders: Official Journal of the Movement Disorder Society* 24 (10): 1453–60.](http://paperpile.com/b/jYy4u9/Hcskf)

[Young, Alexandra L., Razvan V. Marinescu, Neil P. Oxtoby, Martina Bocchetta, Keir Yong, Nicholas C. Firth, David M. Cash, et al. 2018. “Uncovering the Heterogeneity and Temporal Complexity of Neurodegenerative Diseases with Subtype and Stage Inference.” *Nature Communications* 9 (1): 4273.](http://paperpile.com/b/jYy4u9/uwQrI)
